# Dual Inhibitors of c-MET and EGFR in Triple Negative Breast Cancer: Pharmacophore Modeling and Molecular Dynamics Based in Silico Drug Repositioning

**DOI:** 10.5812/ijpr-164183

**Published:** 2025-10-24

**Authors:** Shadi Abkhiz, Parastoo Tarighi, Homa Azizian

**Affiliations:** 1 Department of Medical Biotechnology, Faculty of Allied Medical Sciences, Iran University of Medical Sciences, Tehran, Iran; 2 Endocrine Research Center, Institute of Endocrinology and Metabolism, Iran University of Medical Sciences, Tehran, Iran; 3 Department of Medicinal Chemistry, School of Pharmacy, Iran University of Medical Science, Tehran, Iran

**Keywords:** c-MET, EGFR, Drug Repositioning, Virtual Screening, Molecular Dynamics Simulation, Triple Negative Breast Cancer, Tyrosine Kinase Inhibitors

## Abstract

**Background:**

The cross-talk between mesenchymal-epithelial transition factor (c-MET) and epidermal growth factor receptor (EGFR) plays a role in breast cancer (BC) progression and resistance to various targeted therapies. Consequently, the simultaneous overexpression of c-MET and EGFR in triple-negative breast cancer (TNBC) is associated with poorer clinicopathological outcomes and increased risks. Despite the development of new c-MET and EGFR inhibitors, the high cost of these drugs makes them inaccessible to most patients.

**Objectives:**

Our study investigated the therapeutic potential of existing drugs by repurposing small molecules against these two receptors.

**Methods:**

A database of 2028 small molecule agents was screened using pharmacophore-based virtual screening protocols. To rank the compounds, Gibbs free binding energies were used to analyze their binding energies and interactions with these two receptors.

**Results:**

It was determined that ARR-4 and ADHHRRR-1 represented the most validated pharmacophore models for c-MET and EGFR, respectively, using receiver operating characteristic (ROC), enrichment factor (EF)1%, and Boltzmann-enhanced discrimination of receiver operating characteristic (BEDROC) scores. As a result, eight small molecules were proposed as potential dual inhibitors of c-MET and EGFR, with pasireotide showing the highest affinity for both. According to our analysis of molecular dynamic simulations, pasireotide, the most energetically favorable compound, is proposed as a dual inhibitor of c-MET and EGFR.

**Conclusions:**

Considering pasireotide's potential to target c-MET and EGFR pathways, our findings provide a strong rationale for its further preclinical validation in the treatment of TNBC. The demonstrated efficacy and safety of pasireotide in this aggressive subtype of cancer can now be evaluated through subsequent studies.

## 1. Background

A global cancer statistics report from 2020 indicates that more than 2.2 million female cancer cases were diagnosed as breast cancers (BCs) (1). The triple-negative breast cancer (TNBC) subclass accounts for 15 - 20% of all BCs and does not express the estrogen receptor (ER), progesterone receptor (PR), or human epidermal growth factor receptor 2 (Her-2). This cancer is characterized by low differentiation, high invasiveness, a propensity for local and distant metastases, poor prognosis, and high recurrence rates, making it challenging to design a targeted agent (2). The TNBCs frequently express higher levels of receptor tyrosine kinases (RTKs), including hepatocyte growth factor (HGF) receptor [mesenchymal-epithelial transition factor (c-MET)] and epidermal growth factor receptor (EGFR) (3, 4). A thorough understanding of c-MET and EGFR signaling, as well as redundant downstream pathways such as PI3K/AKT, STAT, and Ras, is essential (5). A study by Linklater et al. found that TNBC patients who expressed high levels of both c-MET and EGFR had poor survival rates (6). The proto-oncogene MET encodes the membrane tyrosine kinase receptor MET (c-MET), which dimerizes when bound to the ligand HGF secreted by stromal cells. The HGF/MET signaling pathway plays a critical role in cell proliferation, survival, embryogenesis, migration, and invasion. Furthermore, research has consistently proven that c-MET is overexpressed in BC, with TNBC displaying the highest levels (7). Crosstalk between c-MET and membrane receptors such as CD44, plexins, FAS, insulin-like growth factor (IGF)-IR, and the EGFR family provides insight into c-MET activity (8).

The EGFR (also referred to as ErbB1, HER1) is one of the RTKs belonging to the ErbB/HER family that regulates numerous developmental processes through various signaling pathways. A wide range of human cancers are closely associated with the hyperactivation of EGFR signaling caused by point mutations, intragenic deletions, or overexpression (9). There is evidence that up to 72% of patients with basal and TNBC express EGFR, and higher expression correlates with a poorer prognosis in patients with TNBC. The persistence of EGFR phosphorylation in TNBC correlates with TKI resistance, possibly due to MET-EGFR crosstalk (6). Additionally, there is an increased incidence of activating mutations of the EGFR gene in non-small cell lung carcinoma, despite its rarity in BC (10).

In the field of computer-aided drug design, pharmacophore-based techniques are integral and have been widely applied to virtual screening, de novo design, and lead optimization. It is possible to derive pharmacophore models from both receptor-based and ligand-based models, which describe the non-bonded interactions between small molecule ligands and macromolecular targets in an abstract way (11, 12).

## 2. Objectives

This research is therefore aimed at identifying and developing a drug candidate focused on dual inhibition of c-MET and EGFR that can treat TNBC, which we assessed using in silico computation techniques. The uniqueness of this study lies in our examination of a small molecules library. Our findings demonstrate that these molecules can inhibit tumor progression and improve treatment outcomes, making them a significant advance in the development of targeted therapies for TNBC. In this study, the most energetically favorable compound against TNBC was validated and established using the most powerful computational method, which may provide therapeutic insights into cancer. Computational investigation has the main advantage of reducing time, cost, and resources. Additionally, these techniques may reduce the chance of failure during clinical or preclinical trials (13).

## 3. Methods

All the in-silico studies were performed using Maestro version 11.8.012, a software from Schrodinger Inc. The Protein Preparation Wizard (PPW), LigPrep, induced fit docking (IFD), develop pharmacophore hypothesis (PHASE), virtual screening workflow (VSW, GLIDE), molecular mechanics/generalized born surface area (MM-GBSA, Prime), and molecular dynamics (MDs) simulation by Desmond were executed on a computer supported by the Linux Ubuntu x86-64 platform.

### 3.1. Protein Preparation

A crucial step in the following process is using a suitable receptor structure. Human c-MET [Protein Data Bank (PDB) ID: 3DKF, 1.80 Å] and EGFR (PDB ID: 4WKQ, 1.85 Å) were retrieved from the PDB (http://www.rcsb.org) (14, 15).

The PPW panel available in Schrodinger Suite 2018 was used to prepare the protein structures (16). The following are the most important modifications applied to the protein structure at this stage.

1. Removal of all crystallographic water molecules beyond 5 Å, except for essential waters involved in ligand binding and coordination with metal cofactors.

2. Addition of all hydrogen atoms to the structure to define correct tautomeric states.

3. Creation of the lost disulfide bonds in the protein structure.

4. Adjustment of the bond orders and formal charges of ligands.

5. Addition and optimization of missing side chains of residues in the protein's crystal structure by running Prime side-chain prediction and Prime structure refinement jobs.

6. Filling in the missing loops using Prime in the protein structure's PDB file and enhancing the quality of the resulting loops by running a Prime loop refinement job.

Finally, the structures were minimized using the optimized potential for liquid simulations (OPLS-2005) force field. Minimization was done with the default constraint of 0.3 Å of root mean square deviation (RMSD) (17).

### 3.2. Ligand Library Preparation

FDA-approved small molecule drugs were used as ligands in this study. The three-dimensional structure of these drugs was obtained from cheminformatic tools and databases for pharmacology and the e-Drug3D section (https://chemoinfo.ipmc.cnrs.fr/MOLDB/index.php). This database contains 2028 molecular structures with a molecular weight of ≤ 2000 daltons (Da). The LigPrep module (18) was used to prepare the ligands with all the necessary conditions, including modification of torsions and assignment of an appropriate protonation state to ensure good representation during protein-ligand interactions. The OPLS-2005 force field was employed for the minimization method, which is useful for preparing the ligand for future investigation.

### 3.3. Pharmacophore Hypothesis Development

Pharmacophores are composed of steric and electronic features necessary to ensure optimal supramolecular interactions with a specific biological target structure and trigger (or block) its biological response (12). This study used the Schrodinger PHASE module to develop the pharmacophore hypothesis (19). Our study employed a crystal structure of c-MET (PDB code: 3DKF) and EGFR (PDB code: 4WKQ) complexed with a known FDA-approved inhibitor to generate a pharmacophore model. A list of specific inhibitors for c-MET and EGFR receptors is shown in Figures S1 and S2 in the Supplementary File, respectively (20, 21). The structures were prepared with LigPrep to prepare the inhibitors, and the parameters were selected similarly to the ligand preparation step. Pharmacophore hypotheses were developed by selecting multiple ligands for each receptor and setting default features such as hydrogen bond acceptors (A), donor bonds (D), hydrophobic groups (H), functional groups with negative charges (N), functional groups with positive charges (P), and aromatic rings (R) for pharmacophore modeling. Three to seven features were selected for constructing the hypothesis, corresponding to at least 50% of these ligands.

### 3.4. Pharmacophore Model Validation and Screening

Validating our pharmacophore models was essential to determine whether they can accurately predict active compounds (22). Active and decoy compounds were used to validate the pharmacophore model separately for each receptor. Pharmacophore models should be statistically significant and capable of retrieving active compounds from data sets to demonstrate their ability to estimate the activity of new compounds. Active and decoy compounds were extracted from the DUD-E database for target receptors (http://dude.docking.org/targets). The active and decoy c-METs were 244 and 11,433, respectively, while the active and decoy EGFRs were 832 and 35,442, respectively. LigPrep was used to prepare all these compounds under the same conditions as before; then, they were entered into the hypotheses validation section. Receiver operating characteristic curves (ROC) were used to identify valid hypotheses. Based on the ROC value, 1 corresponds to ideal screen performance, and 0.5 corresponds to random behavior (23). The area under the curve (AUC) and enrichment factors (EFs) values were calculated from the ROC to estimate the performance of a classification model. As ROC represents the performance of a classification model, AUC represents how well the model can predict the outcome; a model with a higher AUC value should be more predictive. Models with 100% correct prediction rates have an AUC value of 1, as the value ranges from 0 to 1. Model validation is also supported by early EF at 1, 5, 10, and 100 percent (24). In virtual screening, a high Phase hypo score indicates that the model will likely identify good hits. However, a Boltzmann-enhanced discrimination of receiver operating characteristic (BEDROC) score is used to predict molecular activity (25).

Finally, pharmacophore-based screening was conducted on 2028 approved drugs using the best validation results and pharmacophore hypothesis models through the Phase ligand screening module (Maestro 12.8).

### 3.5. Virtual Screening Workflow

The VSW of Maestro 12.8 was used to screen based on docking techniques and score lead-like compounds to identify potential ligands (26). The docking was carried out using the target receptors (c-MET and EGFR) and the resulting compounds from the pharmacophore-based screening. Three different docking protocols were used: High-throughput virtual screening (HTVS), standard-precision (SP), and extra precision (XP). Additionally, based on the MM-GBSA method used to score glide docking results, compounds with a lower Gibbs free energy (kcal/mol) are considered to have a higher affinity for binding to the active site of the target proteins. The HTVS docking algorithm uses the same docking algorithm as SP, but HTVS reduces the number of intermediate combinations in the docking funnel, and refinement and torsion sampling accuracy are lower. In contrast to SP, XP performs wider sampling and uses a scoring function that is "harder" than the SP GlideScore and requires ligand-receptor shape complementation, which eliminates false positives.

For each combined mode, a maximum of 10 modes were created, with 80% of the best combinations retained, and then the best-scoring modes entered SP docking. Similarly, in the SP stage, a maximum of 10 states for each combined state were created, 80% of the best combinations were retained, and only the highest-scored states proceeded to the XP docking stage. The XP docking protocol and Prime MM-GBSA processing were used to calculate protein-ligand binding energy based on non-repetitive results from the SP step. Initially, three modes were created for each combination mode, and 10% of the best combinations were kept along with the highest-scoring mode. As a final step, the outputs were filtered, and duplicate compounds were eliminated.

### 3.6. Prime Molecular Mechanics/Generalized Born Surface Area Calculation

The MM-GBSA modules (Schrodinger LLC 2018) were used to calculate binding energies (ΔG Bind) (27). According to the following equation: ΔG bind = E complex – [E receptor + E ligand]. The calculated relative free energy, which includes both the ligand and receptor strain energy, is ΔG bind, while E complex is the minimum energy calculated by MM-GBSA, E ligand is the MM-GBSA energy of the ligand after removing it from the complex and allowing it to relax, and E receptor is the MM-GBSA energy of the relaxed protein after separating it from the ligand. MM-GBSA calculations were conducted using the best pose structures generated by XP docking complexes.

### 3.7. Induced Fit Docking Investigation

The IFD method using Glide software (Schrodinger LLC 2018, USA) was applied to predict the side-chain and backbone flexibility of the proposed compound with dual inhibition activity over the active site of c-MET and EGFR. The co-crystallized ligand center of c-MET and EGFR at the active site was used to generate the grid box for IFD calculation. The inner box size was defined as a 10 Å cube, and the outer box size as 22 Å. A maximum of 20 poses with receptor and ligand van der Waals radii of 0.7 and 0.5, respectively, were considered. Residues within 5 Å of both active sites were refined, followed by side-chain optimization. Structures whose Prime energy is more than 30 kcal/mol are eliminated based on XP Glide docking.

### 3.8. Molecular Dynamics Simulation

The MDs simulation was performed in this study utilizing the Desmond v5.3 modules, which are part of the Schrodinger 2018-4 suite's Maestro interface (28). To determine the proper pose for the compounds' MD simulation process, the IFD approach was applied (29). The MD simulation involved solvating protein-ligand complexes with SPC water and positioning the molecules in the middle of an orthorhombic box of appropriate size under periodic boundary conditions. The system was neutralized using counterions and a 0.15 M solution of NaCl to mimic real cellular ionic concentrations. This MD protocol involves three steps: Minimization, pre-production, and production. The system was relaxed, the temperature was gradually raised, and simulations were conducted under the NPT ensemble. To minimize energy, the steepest descent approach was applied for 2500 steps. During the gradual increase in temperature from 0 to 300 K, a small force constant was applied to the enzyme. The MD simulations were conducted under the NPT ensemble (constant pressure 1.01325 bar, constant temperature 300 K), using the Nose-Hoover chain method as the default method. A particle-mesh-based Ewald approach was used to calculate electrostatic long-range forces, with Coulombic forces being cut off at 9.0 Å. The MD simulations of the protein-ligand complex were conducted for 80 ns with data frames stored every 1000 ps. Data was analyzed using RMSD and interaction diagrams (30, 31).

## 4. Results and Discussion

### 4.1. Pharmacophore Model Generation

As described in the methodology section, the PHASE module was used to generate pharmacophore hypotheses. Detailed descriptions of each receptor's pharmacophore features are provided below.

The total number of hypotheses generated for c-MET was twenty, including three and four variants (a list of all is in Table S1 in the Supplementary File). The pharmacophore variants of c-MET, which were combinations of the features, included ARR, AAR, AAAR, and AARR. As was evident, each hypothesis was characterized by at least one ring feature (R) and one acceptor feature (A). This observation suggests that A and R properties played an important role in the binding of the compounds to c-MET.

As a result of hypotheses generation for EGFR, fifty variants out of three and four were generated, and these variants are AHH, HRR, DHR, AHHR, DHHR, DHRR, HHRR, DDHHR, DHHRR, DHHRRR, ADHHRR, DDHHRR, AHHRR, AHHRRR, ADHHRRR, DDHHRRR, DDHHHRR, and DHHHRRR (a list of all items in Table S2 in the Supplementary File). By examining all variants, the (A) acceptor, (D) donor, (H) hydrophobic, and (R) aromatic ring features are shown to be significant for EGFR pharmacophore description.

### 4.2. Pharmacophore Model Validation

Validating a pharmacophore provides valuable information about the potential features of active and inactive compounds, usually based on the interaction between specific proteins and ligands (32). The hypotheses generated were ranked and scored based on the ROC, EF1%, Phase hypo score, and BEDROC (Tables S1 and S2 in Supplementary File). Based on the highest ROC, EF1%, and BEDROC scores, ARR-4 and ADHHRRR-1 were selected as the most validated pharmacophore models for c-MET and EGFR, respectively (Table 1). Figures S3 and S4 in Supplementary File display the computed distances within the developed pharmacophoric characteristics of the best hypothesis for c-MET and EGFR, respectively (Figures S3 and S4 in Supplementary File). Additionally, the resulting models have demonstrated the ability to distinguish true actives from decoy compounds by defining ROCs of 0.90 and 0.81 for c-MET and EGFR, respectively (Figure S3B and S4B in Supplementary File).

**Table 1.** The Best Validated Model Pharmacophore of c-Mesenchymal-Epithelial Transition Factor and Epidermal Growth Factor Receptor

Targets	Model Hypothesis	Phase Hypo Score	EF1%	BEDROC160.9	ROC
**c-MET**	ARR-4	0.15	44.6	0.73	0.9
**EGFR**	ADHHRRR-1	0.36	27.09	0.5	0.81

Abbreviations: EF, enrichment factor; BEDROC, Boltzmann-enhanced discrimination of receiver operating characteristic; ROC, receiver operating characteristic; c-MET, c-mesenchymal-epithelial transition factor; EGFR, epidermal growth factor receptor.

### 4.3. Pharmacophore Screening

A database screening query was created using the proposed pharmacophore model through the Phase ligand screening module (Maestro 12.8) to find better drugs with comparable properties. In this study, potential hit compounds were compiled from a subset of FDA-approved small molecules. Analyzing these initial hits was done by aligning them with the best-generated hypothesis. Several key parameters were calculated to determine the precision of the match between the hypothesis and the database compounds. These parameters included fitness, site, vector, volume, and alignment scores (33).

As a first step, we screened the PHASE database using our best pharmacophore model, ARR-4, to remove molecules that did not have the geometrical features necessary to bind to c-MET structures. Among 2028 molecules, only 513 structures passed the filter. The compounds extracted had fitness scores between 0.628 and 3.000. Fitness scores indicate how well a compound matches the pharmacophore hypothesis. A high fitness score suggests that the hit compounds are likely to be very active.

The PHASE database was also screened for EGFR using the best pharmacophore model, ADHHRRR-1, to remove molecules without the geometrical features required for binding. In this case, 882 structures out of 2028 molecules passed the filter, with fitness scores ranging from -0.040 to 3.000. The 2028 compounds retrieved from the FDA database were successfully screened against the pharmacophore features encompassed within the ARR-4 and ADHHRRR-1 models to define the potential hits for c-MET and EGFR, respectively. Among them, 513 and 882 hits were selected based on pharmacophore-based screening.

### 4.4. Virtual Screening Workflow

The virtual screening VSW module (Maestro 12.8) (34) was used to dock potential small molecules from the resulting compounds of pharmacophore screening based on the computational complexity protocol with three different levels of Glide docking precision: The HTVS, SP, and XP.

All the 513 and 882 hits filtered from the pharmacophore screening were subjected to the HTVS mode of the VSW panel utilizing the previously generated Glide grid of c-MET and EGFR, respectively. As a result of applying HTVS as a fast-scoring function, 278 and 600 compounds with docking scores greater than -6 were docked using the SP method over c-MET and EGFR, respectively, to enhance accuracy. Among them, 100 and 300 molecules with docking scores greater than -7 were analyzed by the more extensive XP mode over c-MET and EGFR to obtain more reliable and accurate results by diminishing the possibilities of false positives. Finally, a total of 49 and 87 compounds resulted from the XP docking calculation over c-MET and EGFR, respectively. Additionally, based on the MM-GBSA method used to score Glide docking results, compounds with lower Gibbs free energy (kcal/mol) are considered to have a higher affinity for binding over the active site of the target proteins. Tables S3 and S4 in Supplementary File show the list of the resulting compounds along with their docking score, Glide energy, and Gibbs free energy based on the MM-GBSA method (Tables S3 and S4 in Supplementary File).

Based on the listed compounds, it is noted that three of the FDA-approved c-MET inhibitors, as shown in Figure S1 in Supplementary File (tepotinib, crizotinib, and capmatinib), and all the FDA-approved EGFR inhibitors defined in Figure S2 in Supplementary File (afatinib, dacomitinib, erlotinib, gefitinib, osimertinib, and icontinib) appear in the final virtual screening output, proving the validity of the computational procedure and indicating the potential prediction of the mentioned screening workflow (Figures S1 and S2 in Supplementary File).

### 4.5. Proposed Dual Inhibitor Over c-Mesenchymal-Epithelial Transition Factor and Epidermal Growth Factor Receptor Active Sites

According to the results listed in Tables S3 and S4 in Supplementary File, there are eight compounds that appear in both tables, providing a common pharmacophore model and high Gibbs free energy over both c-MET and EGFR active sites. These compounds, highlighted in Tables S3 and S4 in Supplementary File, include pasireotide, valrubicin, dacomitinib, riboflavin, crizotinib, mebendazole, phenprocoumon, and tolcapone, with crizotinib and dacomitinib having been previously approved for c-MET and EGFR targets, respectively (Tables S3 and S4 in Supplementary File) (35, 36). Additionally, the supplementary data for this study include a comprehensive Table S5 that outlines the known mechanisms of action and therapeutic indications for all the compounds investigated (Table S5 in Supplementary File). This table provides valuable context and background information on the various small molecule inhibitors evaluated in this research.

Among the compounds with the proposed dual inhibition feature, pasireotide, a six-membered cyclic peptide composed of L-phenylglycyl, D-tryptophyl, L-lysyl, O-benzyl-L-tyrosyl, L-phenylalanyl, and modified L-hydroxyproline residues joined in sequence, with binding energies of -93.79 and -103.93 kcal/mol over c-MET and EGFR, respectively, is considered for further in silico investigation. Pasireotide, a somatostatin analog, is an orphan drug approved in the United States and the European Union for the treatment of Cushing's disease in patients who fail or are ineligible for surgical therapy (37). As a somatostatin analog, pasireotide inhibits insulin-like growth factor-1 (IGF-1) actions directly and indirectly in the mammary gland without causing any menopause-related symptoms. Since both progesterone and E2 require IGF-1 to function, pasireotide partially inhibits estrogen action in the mammary epithelium. The inhibition of IGF-I could affect estrogen-dependent and estrogen-independent pathways, making it possible to treat ER-negative breast tumors as well (38, 39). Moreover, pasireotide was introduced as a BC prevention agent by Kuran et al. in their investigation, which also suggested our study. The investigation revealed certain chemical structures of new compounds in BC prevention (40).

Crizotinib has been approved for treating non-small-cell lung cancer (NSCLC) patients by inhibiting mesenchymal-epithelial transition (MET), ALK, and ROS1 kinases. According to a recent study, this small molecule is also effective in inhibiting the proliferation, migration, and invasion of BC cells (41). Additionally, dacomitinib (PF-00299804) is a second-generation irreversible pan-HER RTK inhibitor approved for the first-line treatment of EGFR-sensitive mutated NSCLC patients (42). It has been discovered that dacomitinib inhibits the proliferation of HER-2-amplified BC cell lines with acquired resistance to trastuzumab and lapatinib (43).

### 4.6. Induced Fit Docking Investigation

A key challenge in computational drug discovery is accurately predicting the flexible ligand-receptor complex because this structure can provide insight into important interactions that drive ligand-receptor binding. An analysis of the interactions between the best-scored resulting hit, pasireotide, and the active sites of c-MET and EGFR receptors was conducted using the IFD docking procedure, along with a comparison with the corresponding approved drugs crizotinib and dacomitinib, respectively.

Hydrogen bonds are thought to result from interactions between hydrogen atoms covalently bound to an electronegative atom and the electronegative elements O, N, and F, which play a crucial role in biological structures and functions. They are essential for life to exist on Earth. A growing body of research suggests that alternative atoms are involved in interactions, which share many of the same characteristics as classical hydrogen bonds. It has been shown that these non-classical hydrogen bonds have profound effects on protein, nucleic acid, and carbohydrate interactions (44).

**Figure 1.**
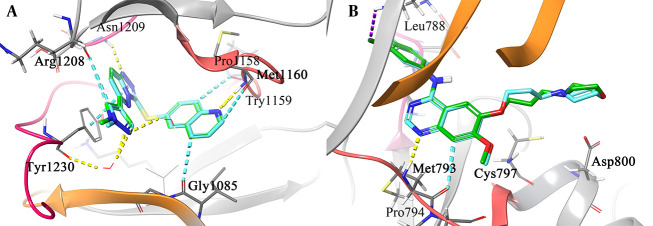
3D depiction of the binding interactions: Docked c-mesenchymal-epithelial transition factor (c-MET) shown in cyan alongside co-crystallized c-MET in green (A) and docked epidermal growth factor receptor (EGFR) in cyan with co-crystallized EGFR in green (B). The structural water and residues responsible for the H-bond interaction are rendered in stick form. The hydrogen bonding, aromatic H-bond, halogen bond, and pi-pi stacking interactions are colored in yellow, faded cyan, violet, and cyan, respectively.

To ensure the accuracy of the docking protocol, each receptor was docked with its respective co-crystallized ligand. The resulting RMSD values of 0.2 Å and 0.5 Å were obtained for the c-MET and EGFR co-crystallized ligands, respectively. This validation step helped optimize the docking accuracy. In Figure 1, the redocked ligands and the co-crystallized ligands are shown superimposed to illustrate their alignment.

As indicated in Figure 1A, the triazolopyridazol ring system of the co-crystallized ligand (SX8) interacts through π-π interaction with Tyr1230 of c-MET, likely playing a critical role in stabilizing the unusual activation loop conformation. The inhibitor's triazolopyridazine moiety forms a classic hydrogen bond with Asp1222, and two non-classic C-H-O=C hydrogen bonds are observed in a bifurcated interaction where pyrazole C-H and triazolopyridazine C-H both move toward the carbonyl oxygen of Arg1208. These interactions further stabilize the system. Furthermore, the quinoline moiety interacts with the c-MET hinge region through a non-classic C-H-O=C link between the nitrogen of the quinoline and the carbonyl oxygen of Pro1158, as well as a canonical hydrogen bond between the nitrogen of the quinoline and the carbonyl of the backbone of Met1160 (14).

Figure 1B illustrates the interaction between gefitinib and Met793 in the EGFR binding pocket via π-π interaction and hydrogen bonds established with the nitrogen atom of the quinazoline ring. Apart from the hydrogen bonding interactions, the aromatic rings of quinazoline and the 3-ethynylphenyl group of gefitinib interact with the aromatic side chain of Gln791 through π-π stacking interactions. By overlaying the delocalized pi-electron systems, these π-π stacking interactions produce an attractive force that aids in anchoring the gefitinib inhibitor within the EGFR active region. As gefitinib binds to EGFR, its 3-chloro-4-fluorophenyl group forms a halogen bond with the carbonyl oxygen of Leu788. As a result of this halogen bond, gefitinib gains additional stabilization and is more likely to attach to the active site of EGFR with higher affinity (15, 45).

Table S5 in Supplementary File represents the free binding energy (kcal/mol) and the interaction of all eight proposed hits through the important active site residues of c-MET. It is observed that all eight hits showed significant interactions with the amino acid residues Met1160 and Pro1158 at the hinge region of the c-MET kinase domain. Additionally, all of them contain moieties that form H-bonds or hydrophobic interactions with the critical amino acid residues Met1211 and Tyr1230 of the activation loop, which are essential for the inhibitory activity of the compounds. Previous studies have demonstrated that the interaction of compounds with Tyr1230 and Met1160 is essential for potent inhibition of c-MET. Furthermore, excellent inhibitory activity at ATP binding sites of the c-MET kinase is associated with the π-π stacked interaction of inhibitors with Tyr1230 or Tyr1230 and Tyr1159 (Table S5 in Supplementary File) (46).

**Figure 2.**
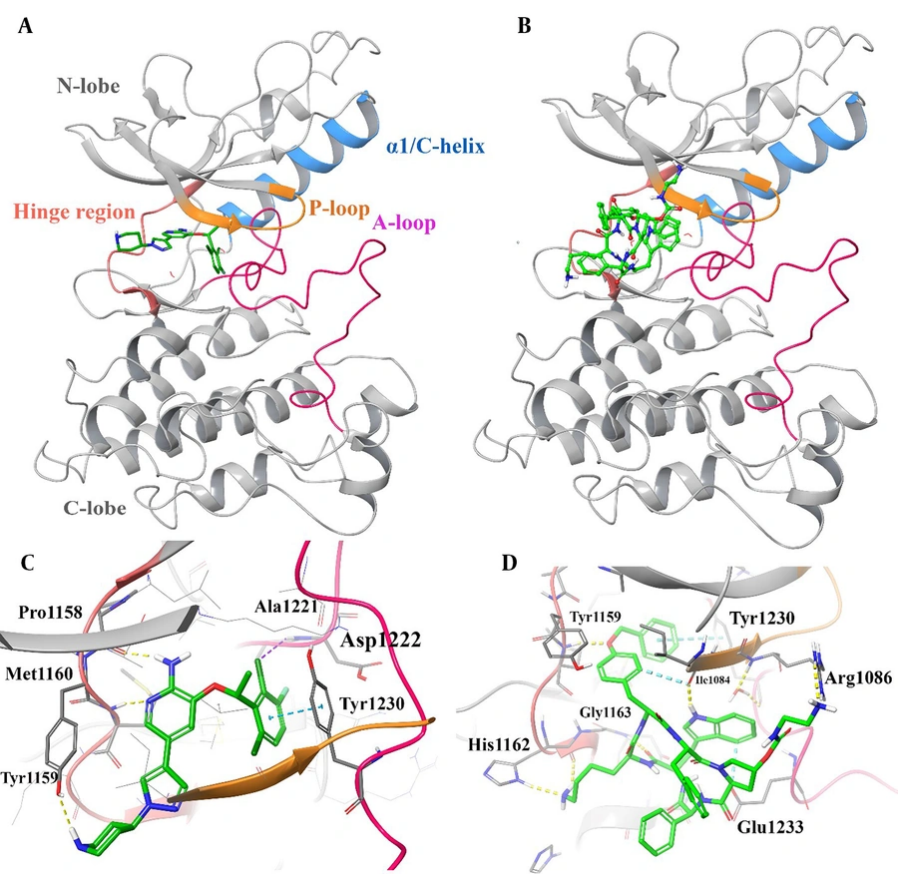
3D representation of crizotinib (A) and pasireotide (B) over the c-mesenchymal-epithelial transition factor (c-MET) kinase domain through the induced fit docking (IFD) procedure along with their corresponding interactions over the relative active site, respectively (C and D). Hydrogen bonding, aromatic H-bond, and pi-pi stacking interactions are colored yellow, cyan, and green, respectively. The hinge region, P-loop, A-loop, and Cα helix are colored in faded red, orange, pink, and blue, respectively.

The 3D representation of the top-ranked hit, "pasireotide", over the active site of c-MET was employed to depict all the essential interactions in comparison to crizotinib as an approved c-MET inhibitor (Figure 2). Based on the observation, pasireotide does indeed bind to the ATP site of the c-MET active site, similar to crizotinib, which is surrounded by the hinge region (residues 1157 - 1164), P-loop (residues 1085 - 1090), A-loop (residues 1221 - 1251), and Cα helix (residues 1062 - 1131; Figure 2A and B).

Based on Figure 2C, the halogenated phenyl moiety of crizotinib adopts a direct and favorable orientation to establish a π-π interaction with Tyr1230, and the 2-chloro and 3-fluoro substituents on the 3-benzyloxy group of crizotinib are oriented towards Asp1222, suggesting the potential for favorable electrostatic interactions. The importance of Tyr1230 in the interaction with crizotinib in c-MET explains the higher potency of the FDA-approved crizotinib (47). Additionally, the plane of the 2-aminopyridine core in crizotinib forms an H-bond interaction with the hinge region through residues Pro1158 and Met1160. Furthermore, the 5-pyrazolyl group is bound through the narrow lipophilic tunnel surrounded by Ile1084 and Tyr1159.

As observed in Figure 2D, the N-(2-aminoethyl) carbamate moiety of the L-pyrolydyl side chain is stabilized through Arg1086 at the beginning of the P-loop. Moreover, the O-benzyl side chain group forms a T-shaped hydrophobic interaction with Tyr1230 located at the c-MET A-loop, an H-bond interaction with Tyr1159 and Ile1084 through its terminal phenyl ring moiety, the O atom, and internal phenyl ring, respectively. Additionally, the D-tryptophyl side chain group of the cyclic system is pinioned between the hydrophobic side chains of Ile1084 and Glu1233 through H-bond and non-classical H-bond interactions, respectively, which stabilize the A-loop. The L-lysyl side chain group of pasireotide forms two H-bond interactions with His1162 (ring -NH) and Gly1163 (NH backbone) of the hinge region through its -NH2 group. Additionally, the cyclic backbone of pasireotide (C=O) interacts with the hinge residue Gly1163 through H-bond interaction. According to the presented results, the interactions made by the L-lysyl moiety closely resemble interactions formed by the 2-aminopyridine core of the ATP-competitive kinase inhibitor, crizotinib, at the hinge region of c-MET.

Moreover, Table S6 in Supplementary File presents the free binding energy (kcal/mol) and the interactions of all eight hits with the key active site residues of EGFR. It was observed that all eight hits exhibited significant interactions with the amino acid residues Leu718 and Val726 at the P-loop, and Ala743, Leu844, and Met793 at the hinge region of the EGFR kinase domain. Additionally, each hit contains a moiety capable of forming hydrogen bonds or hydrophobic interactions with the critical amino acid residue, Cys797, of the extended hinge region of the kinase domain, which is essential for the inhibitory activity of these compounds. Based on the results, Met793, located within the hinge region, is involved in the binding of all the proposed drugs. Additionally, Asp800, situated in the A-loop region, can also facilitate ligand binding to the receptor in crizotinib, pasireotide, and valrubicin. Therefore, it is proposed that the hinge and A-loop regions are crucial for these interactions (Table S6 in Supplementary File).

An investigation conducted by Zhao et al. revealed that there are 39 residues close to the ATP-binding pocket, arranged in β-sheets, hinge regions, and α-helix, in which the most frequent contact is found at residues Leu718, Val726, Ala743, Met793, and Leu844, which form a hydrophobic core binding pocket that is conserved in the crystal structure of the EGFR kinase domain (48). Furthermore, as a result of Balogun et al., residues consisting of Met793, Lys745, Phe723, Asp855, Arg411, and Thr854 were found to be the principal amino acid residues involved in EGFR-ligand interactions (49).

**Figure 3.**
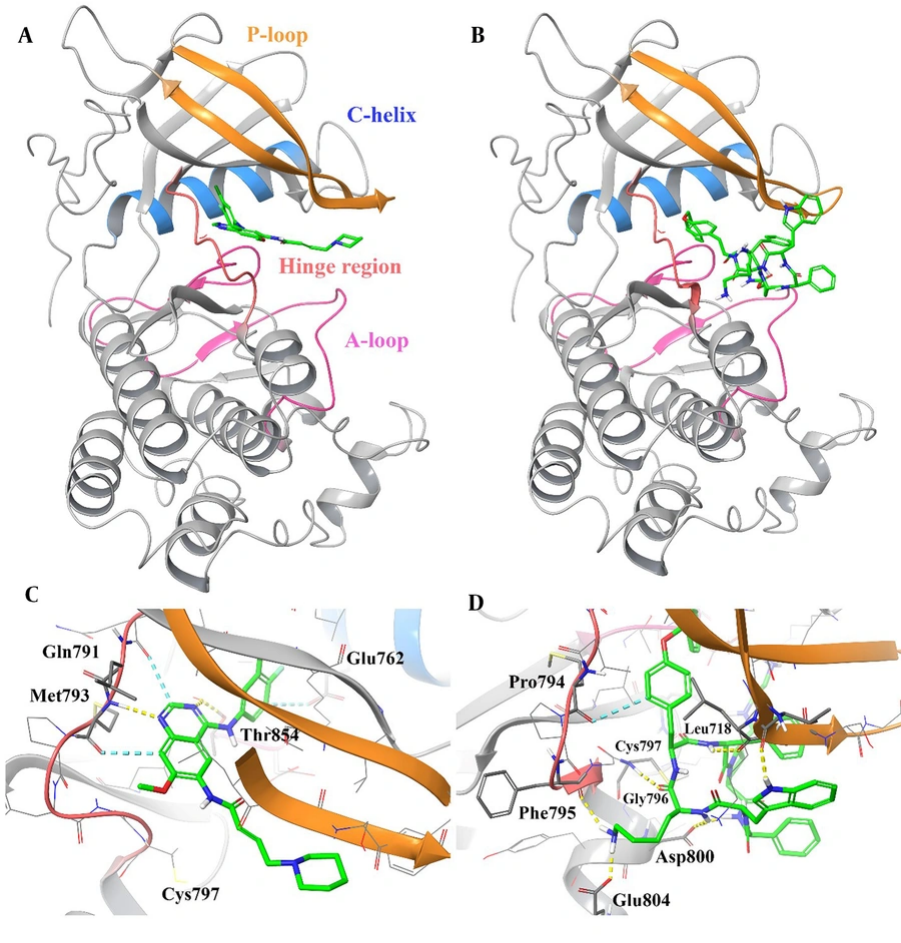
3D representation of dacomitinib (A) and pasireotide (B) over the epidermal growth factor receptor (EGFR) kinase domain through the induced fit docking (IFD) procedure along with their corresponding interactions over the relative active site, respectively (C and D). Hydrogen bonding, aromatic H-bond, and pi-pi stacking interactions are colored yellow, cyan, and green, respectively. The hinge region, P-loop, A-loop, and Cα helix are colored in faded red, orange, pink, and blue, respectively.

The 3D representation of the top-ranked hit, "pasireotide", over the active site of EGFR was employed to depict all the essential interactions in comparison to dacomitinib as an approved EGFR inhibitor (Figure 3). 

Based on the observations, pasireotide binds to the ATP site of the EGFR active site similarly to dacomitinib, which is surrounded by the hinge region (residues 791 - 797), P-loop (residues 712 - 731), A-loop (residues 854 - 882), and Cα helix (residues 756 - 768; Figure 3A and B).

Based on Figure 3C, the plane of the 7-methoxyquinazoline core in dacomitinib is stabilized at the hinge region by forming an H-bond interaction with Met793 (-NH group) and a non-classical H-bond interaction with Gln791 and Met793. The mentioned core forms an H-bond interaction with Thr854 at the beginning part of the A-loop. Additionally, the 3-chloro-4-fluorophenyl group moiety of dacomitinib adopts a non-classical H-bond with Glu762 at the Cα helix part around the active site.

As observed in Figure 3D, the internal phenyl of the O-benzyl side chain and the D-tryptophyl side chain group of the cyclic system are oriented toward the EGFR hinge region, which is stabilized by H-bond and non-classical H-bond interactions with Gly796 and Gly719, respectively. Also, the D-tryptophyl side chain group of the cyclic system is located between the hydrophobic side chains of Ile084 and Glu1233 through H-bond and non-classical H-bond interactions, respectively, which stabilize the A-loop. The L-lysyl side chain group of pasireotide forms two H-bond interactions with Phe795 (backbone C=O) at the hinge region and Glu804 (side chain COO). Additionally, the cyclic backbone of pasireotide (-NH) interacts with Leu718 and Asp800 through H-bond interactions. These results show that the overall binding orientation of pasireotide is very similar to the binding mode of dacomitinib at the EGFR kinase domain.

The previous study implies that the electrophilic moiety of dacomitinib is subjected to nucleophilic attack by Cys797 of the extended hinge region of the kinase domain, forming a covalent bond between the two, which provides irreversible inhibition at the ATP binding pocket (50). According to the IFD study, Figure S5A in Supplementary File illustrates the orientation of Cys797 relative to the (piperidin-1-yl) but-2-enoyl tail of dacomitinib, suggesting its potential to facilitate a Michael-addition reaction (Figure S5A in Supplementary File).

In the case of pasireotide, the C=O moiety belonging to the amide group of L-hydroxyproline residues is the most susceptible amide to enhance the intrinsic electrophilic reactivity against Cys797 of EGFR. As observed in Figure S5B in Supplementary File, the spatial orientation of the modified L-hydroxyproline moiety on the cyclic ring of pasireotide, facing towards Cys797, indicates the possibility of a Michael-addition reaction at this site, akin to that observed in the complex structures with dacomitinib. It is reported that an amide bond with a higher proton affinity may be more readily opened due to the basicities of the amide nitrogen (51). This is the case with proline-containing cyclic peptides when collision-activated under energy conditions; they afford selectivity by undergoing selective ring cleavage at the proline residue (Figure S5B in Supplementary File) (52).

Moreover, the proposed reaction mechanism of Cys797 alkylation for acrylamide in dacomitinib and the susceptible amide bond in pasireotide at the ATP binding pocket of EGFR, as defined in Figures S5C and S5D, respectively, offers important information about the chemical interactions and potential covalent changes between critical cysteine residues in the EGFR active site and small molecule inhibitors (Figures S5C and S5D in Supplementary File).

Dacomitinib's acrylamide moiety alkylates Cys797, a well-studied covalent binding process that increases the EGFR inhibitor's effectiveness and selectivity. A stable covalent adduct can be formed between the electrophilic acrylamide group and the nucleophilic thiol side chain of Cys797 through a Michael addition process. This irreversible attachment to the ATP binding site inhibits EGFR kinase activity and effectively blocks ATP access.

Regarding pasireotide, the sensitive amide bond in the molecule is located near Cys797 in the EGFR active region, as illustrated in Figure S5D in Supplementary File. The amide bond has the potential to generate a covalent link between pasireotide and the cysteine residue through hydrolysis or nucleophilic attack. Such covalent changes can alter the binding affinity and inhibitory potency of pasireotide towards EGFR.

### 4.7. Molecular Dynamics Simulation Studies

The MD simulation analysis was carried out on pasireotide based on its favorable binding affinity compared to other hit compounds. Its interaction profile suggests that it could effectively inhibit the active sites of c-MET and EGFR. To investigate the stability of the modeled systems, the dynamic behavior of the optimal IFD pose of pasireotide at c-MET and EGFR active sites was compared with the dynamic action of crizotinib and dacomitinib, the standard inhibitors of the c-MET and EGFR kinase domains, respectively, over approximately 80 ns of MD simulation to predict the motion of complex systems at an atomistic level. The RMSD values serve as indicators of the system’s conformational stability and perturbations (53).

Monitoring the RMSDs of the protein and the best-scored hit, pasireotide, along with the standard inhibitors of c-MET and EGFR, was carried out throughout the entire simulation time. During the 80 ns simulation, 1000 frames were captured every 1 ps. The collection of frames stored as a trajectory provides valuable information about the structural conformation throughout the interaction. It also indicates the stability of the interaction and whether the simulation has reached equilibrium.

According to the RMSD plots, all the systems reached an equilibrated level and exhibited consistent stability within the c-MET active site. Consequently, it can be concluded that the c-MET structure is not significantly altered by the ligand-protein complex. The RMSD average values for c-MET in non-bonded form and in complex with crizotinib and pasireotide were 3.1, 2.1, and 2.6 Å, respectively. The crizotinib-c-MET complex demonstrated a lower deviation value compared to the pasireotide-c-MET complex and the non-bonded form, indicating higher stability of the crizotinib-c-MET complex. Additionally, the pasireotide-c-MET complex exhibited a smaller RMSD value than the non-bonded form, which implies the formation of a stable complex between pasireotide and c-MET upon the formation of favorable interactions with key residues.

**Figure 4.**
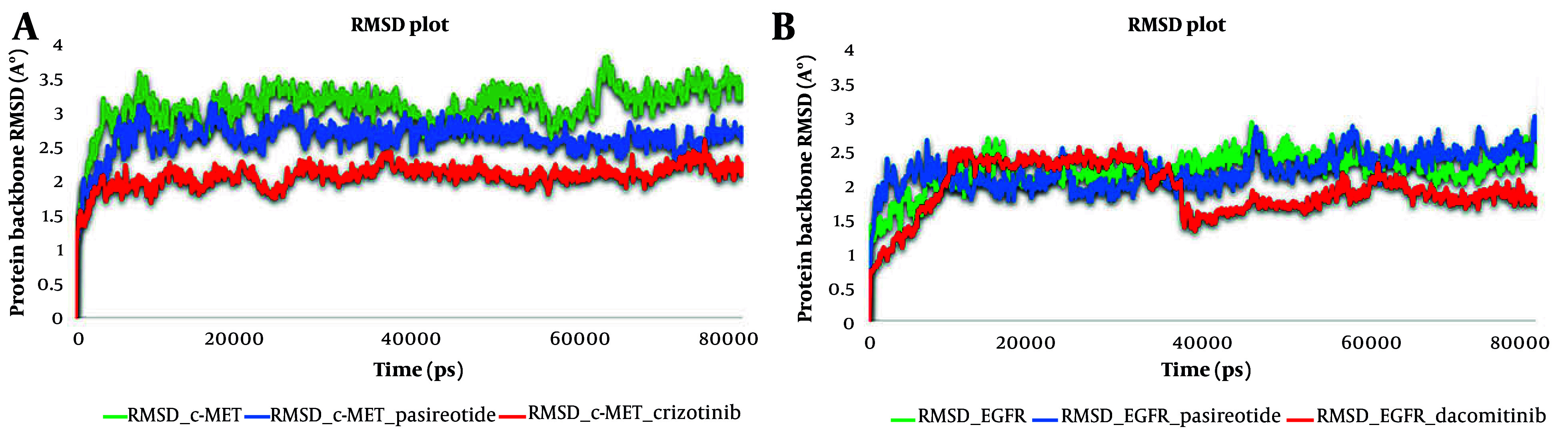
Root mean square deviation (RMSD) of the c-mesenchymal-epithelial transition factor (c-MET) kinase domain in complex with pasireotide and crizotinib and in non-bonded form (A), and the RMSD of the epidermal growth factor receptor (EGFR) kinase domain in complex with pasireotide and dacomitinib and in non-bonded form (B) over 80 ns molecular dynamic (MD) simulation time.

Furthermore, Figure 4 illustrates the stability of the dynamic behavior of EGFR in complex with pasireotide and dacomitinib, as well as in its non-bonded form, with RMSD average values of 2.2, 2.9, and 2.2 Å, respectively. In the EGFR-dacomitinib complex, equilibrium was achieved after 40 ns. A stable equilibrium was achieved at around 10 ns for EGFR-pasireotide, with fluctuations remaining around the RMSD value of 2.5 Å after 40 ns. The RMSD simulation showed that the pasireotide-EGFR complex and the non-bonded system remained stable throughout the 80 ns simulation. Such observations indicated that the employed simulation time was sufficient to obtain an equilibrium structure over the simulation period.

Root mean square fluctuation (RMSF) values of the protein’s residues were also analyzed to demonstrate its flexibility and determine where a protein's structure fluctuates compared to its overall structure. Loosely arranged loops tend to have higher RMSF values than those with sheets and helices. A lower RMSF value signifies enhanced system stability, whereas a high RMSF value denotes increased flexibility throughout the MDs simulation.

Figure S6A in Supplementary File indicates that the RMSF values for the P-loop (1085 - 1090), α-helix (1062 - 1131), hinge region (1157 - 1164), and the outer A-loop (1275 - 1300) of c-MET in the non-bonded state are about 2.4, 2, 1, and 2.2 Å, respectively. Figures S6B and S6c show the overall RMSF similarity of the bonded state domain, where the RMSF of the P-loop decreases to 0.7 and 1.2 Å, the α-helix decreases to 1.5 and 1.4 Å, the hinge region decreases to 0.7 and 0.9 Å, and the outer A-loop decreases to 1.8 and 1.5 Å in c-MET complexed with crizotinib and pasireotide, respectively (Figure S6A and S6B in Supplementary File).

By comparing the RMSF plot, it is revealed that important regions of the c-MET kinase domain, such as the P-loop, α-helix, hinge region, and A-loop, are more stabilized in the crizotinib-c-MET and pasireotide-c-MET complexes than in the non-bonded state. An investigation conducted by Collie et al. indicated that selectively targeting the folded P-loop conformation may enhance the effectiveness and selectivity of kinase inhibitors (54). Additionally, pasireotide, like crizotinib, interacted with the hinge region and extensively with the A-loop (colored in green line), consequently affecting the overall stability of the c-MET domain.

Figure S7A in Supplementary File indicates that the RMSF values for the EGFR P-loop (712 - 731), α-helix (756 - 768), hinge region (791 - 797), and the outer A-loop (904 - 934) in the non-bonded state are about 2.4, 2.8, 0.8 - 1, and 4.2 Å, respectively. Figures S7B and S7C in Supplementary File show the overall RMSF similarity of the EGFR bonded state domain in complex with dacomitinib and pasireotide, where the RMSF of the P-loop decreases to 1.6 and 2 Å, the α-helix decreases to 1.5 and 1.4 Å, the hinge region decreases to 0.7 and 0.9 Å, and the outer A-loop decreases to 1.8 and 1.5 Å in c-MET complexed with crizotinib and pasireotide, respectively (Figure S7A - C in Supplementary File).

By comparing the RMSF plot, it is revealed that important regions of the c-MET kinase domain, such as the P-loop, α-helix, hinge region, and A-loop, are more stabilized in the crizotinib-c-MET and pasireotide-c-MET complexes than in the non-bonded state. An investigation conducted by Collie et al. indicated that selectively targeting the folded P-loop conformation may enhance the effectiveness and selectivity of kinase inhibitors (54). Additionally, pasireotide, like crizotinib, interacted with the hinge region and extensively with the A-loop (colored in green line), consequently affecting the overall stability of the c-MET domain.

Considering the importance of the inhibitors' interactions with the hinge binding region and gatekeeper, RMSF values in this region are quite low (55). According to other articles, the intracellular domain of EGFR contains seven helices and seven sheets, and an inhibitor can compete with the natural substrate for ATP-binding sites by being locked into a "mouth-like" structure. The P-loop, helix, hinge region, and A-loop make up the groove in the mouth (56).

The 2D interaction diagram of crizotinib and pasireotide in complex with c-MET is depicted in Figure S8A and S8C in Supplementary File, showing various residues and types of interactions that occurred for at least 30% of the entire MD simulation time. According to Figure S8A in Supplementary File, the aminopyridine moiety of crizotinib was stabilized at the c-MET active site opening space through hydrogen-bonding interactions with Pro1158 and Met1161 for about 99% and 67% of the MD simulation, respectively. Additionally, the halogenated benzyl group was stabilized via hydrophobic π–π stacking interaction by the Tyr1230 residue during approximately 87% of the MD simulation time. Based on the timeline result, crizotinib interacts continuously with Ile1084 at the P-loop, Ala1108 at the α-helix part, Pro1158 and Met1160 at the hinge region, as well as Tyr1230 and Met1211 at the A-loop of the c-MET active site (Figure S8A and S8B in Supplementary File).

As observed in Figure S8C in Supplementary File, the N-(2-aminoethyl) carbamate moiety of the L-pyrolydyl side chain is stabilized through Glu1233 for about one-third of the simulation time. Additionally, the D-tryptophyl side chain group (-NH) of the cyclic system is oriented toward Asn1167 through a water-mediated H-bond for about 34% of the simulation time, stabilizing the A-loop. The L-lysyl side chain group of pasireotide formed water-mediated H-bond interactions with Ile1084 (α-helix) through its -NH2 group for about 40% of the MD simulation time. Furthermore, the cyclic backbone of pasireotide (C=O) interacted with Arg1086 (α-helix), Lys1232 (A-loop), and His1162 (hinge region) through water-mediated H-bond interactions for about 33%, 70%, and 32% of the simulation time, respectively (Figure S8C in Supplementary File).

The interaction timeline representation shows that throughout the majority of the MD simulation time, pasireotide facilitated interactions through residues Val1083, Ile1084, and Arg1086, which are coordinated near and at the beginning of the P-loop of the active site. Additionally, consistent interactions with Met1160 and Asp1164 at the hinge region, and Tyr1230, Lys1232, and Glu1233 at the A-loop, were observed during the MD simulation time (Figure S8D in Supplementary File).

Figure S9 in Supplementary File depicts the molecular interactions of pasireotide and dacomitinib over the EGFR active site. As shown in Figure S9A in Supplementary File, dacomitinib formed H-bond and water-mediated H-bond interactions with Met793 and Arg841 for about 95% and 35% of the MD equilibrated phase, respectively. During the equilibrated phase of the MD simulation, pasireotide produced all interactions through water-mediated H-bonds with Leu717, Cys797, and Asp800 for about 30%, 53%, and 31% of the MD simulation time, respectively (Figure S9A and S9C in Supplementary File).

Dacomitinib facilitated interactions through residues Leu718, Ala743, Met793, and Leu844, which were coordinated at the center of the active site for the duration of the MD simulation, as shown by the interaction timeline representation (Figure S9D in Supplementary File). While the simulation process continued, certain interactions — such as with Ser720 — diminished, and new interactions — like those with residues Phe723 and Cys797 — arose, producing interactions that remained stable for the duration of the simulation. Pasireotide forms stronger connections with the residues Asp800, Cys797, and Leu718 compared to dacomitinib. Additionally, as seen in Figure S9D in Supplementary File, pasireotide interacted with Phe723, Val726, Ala743, Leu747, Glu804, and Arg841 during the MD simulation period (Figure S9D in Supplementary File).

Moreover, the hinge region (Gln791 to Leu798), which makes up the inhibitors' binding functional areas (56), is primarily present in these residues and has been shown to be relatively flexible and significant in many complexes.

To conduct a more in-depth investigation into the probable mechanism of pasireotide over the EGFR active site, based on the proposed mechanism shown in Figures S8C and S8D, two measurements were calculated. The first was the distance of the carbon of the C=O moiety belonging to the amide group of L-hydroxyproline residues, which is recognized as the most susceptible amide to enhance the intrinsic electrophilic reactivity against Cys797 of EGFR (Figure S10A in Supplementary File). The second measurement was the distance between Cys797 (-SH) as a nucleophile and Asp800 (COO^-^) as a catalytic base (Figure S10B in Supplementary File), compared with the corresponding distance in the dacomitinib-EGFR complex (Figure S10A and S10B in Supplementary File).

Figure S10A in Supplementary File displays the distance of the carbon of the C=O moiety belonging to the amide group of L-hydroxyproline residues, which is recognized as the most susceptible amide to enhance the intrinsic electrophilic reactivity against Cys797 of EGFR. It is evident that the mentioned distance in the pasireotide-EGFR complex fluctuated around 5 Å, which is lower than that in the dacomitinib-EGFR complex (approximately 6 Å) for about the first half of the MD simulation time. For the rest of the simulation time, the mentioned distance increased and finally reached 7 Å, the same as in the dacomitinib-EGFR complex.

Additionally, Figure S10B in Supplementary File shows that the distance between Cys797 (-SH) as a nucleophile and Asp800 (COO^-^) as a conjugated base in the pasireotide-EGFR complex during the first half of the MD simulation time is lower than that of the dacomitinib-EGFR complex, with quantities around 4 Å and 6 Å, respectively. Again, the mentioned distance increased and then equilibrated to the corresponding length in the dacomitinib-EGFR complex.

Based on the reported results and the proposed mechanism mentioned in Figure S5D in Supplementary File, the proper spatial orientation and distance of the modified L-hydroxyproline moiety on the cyclic ring of pasireotide, which faces towards Cys797, indicates the possibility of a Michael-addition reaction at this site, akin to that observed in the complex structures with dacomitinib. In summary, the closer proximity of Cys797 (-SH) to the susceptible electrophilic moiety and to Asp800 increases the probability of Cys797 alkylation through a Michael-addition reaction.

Several studies targeting c-MET and EGFR in TNBC have been conducted; however, they primarily focus on experimental approaches (6, 57), whereas our study employs pharmacophore-based virtual screening as a novel approach in this context. Moreover, the in-silico studies we referenced were related specifically to lung cancer, not TNBC.

### 4.8. Prime/Molecular Mechanics/Generalized Born Surface Area Calculation

The Prime/MM-GBSA module was utilized to assess the strength of interactions between ligands and proteins in addition to the interaction analysis, and this was done throughout MD. As shown in Table S7 in Supplementary File, the ΔGbind for the crizotinib-c-MET complex and pasireotide-c-MET complex were reported. The negative ΔGbind values indicate that these ligands are thermodynamically favorable and spontaneously bind to the c-MET kinase. Furthermore, compared to pasireotide, dacomitinib has a greater binding affinity for EGFR (Table S7 in Supplementary File).

In addition to their potential therapeutic uses, these discoveries may have significant implications for kinase inhibitors. Pasireotide may be a more potent and effective inhibitor of both oncogenic kinases when employed in cancer treatment since it has higher binding affinities with both EGFR and c-MET kinases, as shown by its more favorable ΔGbind values. Notably, the ΔGbind values offer a quantitative approximation of the binding free energy, accounting for many elements such as solvation effects, van der Waals interactions, and electrostatic interactions. A ligand-target protein binding relationship that is more persistent and advantageous is typically indicated by a greater negative ΔGbind value.

It would be necessary to conduct more experimental and clinical research to identify the therapeutic potential of these kinase inhibitors and validate the computational predictions derived from the Prime/MM-GBSA analysis. As a result of this study, we gained a greater understanding of the molecular mechanisms that govern these inhibitors' interactions with their target kinases, which may also help develop and refine new therapeutic medicines for cancer.

### 4.9. Conclusions

To discover and repurpose small molecules for dual inhibition of c-MET and EGFR, a set of computational methods was performed through a database of 2028 FDA-approved small molecules over both c-MET and EGFR kinase domain active sites. Compounds' binding modes and energies were analyzed and ranked. Accordingly, eight agents, including pasireotide, valrubicin, dacomitinib, riboflavin, crizotinib, mebendazole, phenprocoumon, and tolcapone, are proposed as potential dual inhibitors of these two receptors. However, further investigation is strongly recommended to assess the effectiveness and capabilities of these compounds.

To summarize, in silico studies were conducted on pasireotide to gain insight into its interaction with both active sites. According to the observations, pasireotide binds to the ATP site of c-MET's active site, just as crizotinib does, and it binds to EGFR's ATP site just as dacomitinib does. Furthermore, RMSDs were tracked during the simulation on the protein and the top hit, pasireotide, as well as conventional inhibitors of EGFR and c-MET. The RMSD plots indicated that all systems reached a stable equilibrium in c-MET and EGFR. Notably, the RMSD value of the pasireotide-c-MET complex was lower than that of the non-bonded form, suggesting the creation of a stable complex between pasireotide and c-MET due to advantageous interactions with important residues. Both the pasireotide-EGFR complex and the non-bonded system maintained their stability throughout the entire 80 ns of the MD simulation time. These observations were validated by residue interactions monitored in RMSF, which demonstrated that the most significant residues in inhibitory roles participate in pasireotide interaction in the active sites of c-MET and EGFR.

Additionally, the Prime MM-GBSA method was used to calculate the binding free energy of pasireotide and authorized inhibitors in their complex with c-MET and EGFR. Stronger ligand-receptor binding is shown by MM-GBSA analysis. As a result of the study on pasireotide, it has been determined that it can be proposed as an effective inhibitor for both c-MET and EGFR.

Virtual screening of FDA-approved small molecules against c-MET and EGFR identified top hits. Pasireotide, a somatostatin analog, emerged as the best candidate based on binding affinities. In silico docking revealed Pasireotide's ability to occupy the ATP-binding pockets of c-MET and EGFR. Molecular dynamics simulations confirmed Pasireotide's stable binding to the active sites of c-MET and EGFR.

### 4.10. Limitations

It is important to recognize that pharmacophore modeling is a valuable tool in drug discovery and design. However, it comes with limitations that can affect virtual screening results, potentially resulting in false positives and false negatives. As a result, it is crucial to remain mindful of these limitations and seek appropriate solutions whenever possible, maintaining an open-minded perspective as we analyze the results. Furthermore, in silico studies must be validated through experimental verification, which was beyond the scope of our investigation.

ijpr-24-1-164183-s001.pdf

## Data Availability

The authors confirm that the data supporting the findings of this study are available within the article and supplementary materials, and also from the corresponding author upon reasonable request.

## References

[1] Muhammad N, Hanif M, Yang P (2024). Beyond cisplatin: New frontiers in metallodrugs for hard-to-treat triple negative breast cancer.. Coordination Chemistry Reviews..

[2] Liu Y, Hu Y, Xue J, Li J, Yi J, Bu J (2023). Advances in immunotherapy for triple-negative breast cancer.. Molecular Cancer..

[3] Corkery B, Crown J, Clynes M, O'Donovan N (2009). Epidermal growth factor receptor as a potential therapeutic target in triple-negative breast cancer.. Ann Oncol..

[4] Khetan D, Verma A, Chaudhary RK, Shukla JS (2020). Molecular characterisation of RhD variants in North Indian blood donor population.. Transfus Med..

[5] Lefebvre C, Allan AL (2021). Anti-proliferative and anti-migratory effects of EGFR and c-Met tyrosine kinase inhibitors in triple negative breast cancer cells.. Precision Cancer Medicine..

[6] Linklater ES, Tovar EA, Essenburg CJ, Turner L, Madaj Z, Winn ME (2016). Targeting MET and EGFR crosstalk signaling in triple-negative breast cancers.. Oncotarget..

[7] Jabbarzadeh Kaboli P, Chen HF, Babaeizad A, Roustai Geraylow K, Yamaguchi H, Hung MC (2024). Unlocking c-MET: A comprehensive journey into targeted therapies for breast cancer.. Cancer Lett..

[8] Fu J, Su X, Li Z, Deng L, Liu X, Feng X (2021). HGF/c-MET pathway in cancer: from molecular characterization to clinical evidence.. Oncogene..

[9] Bi J, Wu Z, Zhang X, Zeng T, Dai W, Qiu N (2023). TMEM25 inhibits monomeric EGFR-mediated STAT3 activation in basal state to suppress triple-negative breast cancer progression.. Nat Commun..

[10] Kim A, Jang MH, Lee SJ, Bae YK (2017). Mutations of the Epidermal Growth Factor Receptor Gene in Triple-Negative Breast Cancer.. J Breast Cancer..

[11] Seidel T, Schuetz DA, Garon A, Langer T (2019). The Pharmacophore Concept and Its Applications in Computer-Aided Drug Design.. Prog Chem Org Nat Prod..

[12] Giordano D, Biancaniello C, Argenio MA, Facchiano A (2022). Drug Design by Pharmacophore and Virtual Screening Approach.. Pharmaceuticals (Basel)..

[13] Kalhor H, Rahimi H, Akbari Eidgahi MR, Teimoori-Toolabi L (2020). Novel Small Molecules against Two Binding Sites of Wnt2 Protein as potential Drug Candidates for Colorectal Cancer: A Structure Based Virtual Screening Approach.. Iran J Pharm Res..

[14] Buchanan SG, Hendle J, Lee PS, Smith CR, Bounaud PY, Jessen KA (2009). SGX523 is an exquisitely selective, ATP-competitive inhibitor of the MET receptor tyrosine kinase with antitumor activity in vivo.. Mol Cancer Ther..

[15] Yosaatmadja Y, Squire C, McKeage C, Flanagan M (2014). 1.85 angstrom structure of EGFR kinase domain with gefitinib.. Protein Data Bank. Available online: https://www. rcsb. org/structure/4WKQ (accessed on 15 August 2022)..

[16] Schrödinger Release 2021-3 (2016). Protein preparation wizard..

[17] Hatami S, Sirous H, Mahnam K, Najafipour A, Fassihi A (2023). Preparing a database of corrected protein structures important in cell signaling pathways.. Res Pharm Sci..

[18] Schrödinger Release (2018). LigPrep, Schrödinger..

[19] Phase (2018). Schrödinger..

[20] Damghani T, Elyasi M, Pirhadi S, Haghighijoo Z, Ghazi S (2022). Type II c-Met inhibitors: molecular insight into crucial interactions for effective inhibition.. Mol Divers..

[21] Zubair T, Bandyopadhyay D (2023). Small Molecule EGFR Inhibitors as Anti-Cancer Agents: Discovery, Mechanisms of Action, and Opportunities.. Int J Mol Sci..

[22] Fei J, Zhou L, Liu T, Tang XY (2013). Pharmacophore modeling, virtual screening, and molecular docking studies for discovery of novel Akt2 inhibitors.. Int J Med Sci..

[23] Truchon JF, Bayly CI (2007). Evaluating virtual screening methods: good and bad metrics for the "early recognition" problem.. J Chem Inf Model..

[24] Opo F, Rahman MM, Ahammad F, Ahmed I, Bhuiyan MA, Asiri AM (2021). Structure based pharmacophore modeling, virtual screening, molecular docking and ADMET approaches for identification of natural anti-cancer agents targeting XIAP protein.. Sci Rep..

[25] Marondedze EF, Govender KK, Govender PP (2020). Ligand-based pharmacophore modelling and virtual screening for the identification of amyloid-beta diagnostic molecules.. J Mol Graph Model..

[26] Birjandi AA, Suzano FR, Sharpe PT (2020). Drug Repurposing in Dentistry; towards Application of Small Molecules in Dentin Repair.. Int J Mol Sci..

[27] Aier I, Semwal R, Sharma A, Varadwaj PK (2021). In silico identification of therapeutic compounds against microRNA targets in drug-resistant pancreatic ductal adenocarcinoma.. J Biomol Struct Dyn..

[28] Schrodinger (2023). Desmond Molecular Dynamics System, Maestro-Desmond Interoperability Tools..

[29] Azimi F, Azizian H, Najafi M, Khodarahmi G, Saghaei L, Hassanzadeh M (2021). Design, synthesis, biological evaluation, and molecular modeling studies of pyrazole-benzofuran hybrids as new alpha-glucosidase inhibitor.. Sci Rep..

[30] Sherafati M, Mirzazadeh R, Barzegari E, Mohammadi-Khanaposhtani M, Azizian H, Sadegh Asgari M (2021). Quinazolinone-dihydropyrano[3,2-b]pyran hybrids as new alpha-glucosidase inhibitors: Design, synthesis, enzymatic inhibition, docking study and prediction of pharmacokinetic.. Bioorg Chem..

[31] Hamedifar H, Mirfattahi M, Khalili Ghomi M, Azizian H, Iraji A, Noori M (2024). Aryl-quinoline-4-carbonyl hydrazone bearing different 2-methoxyphenoxyacetamides as potent alpha-glucosidase inhibitors; molecular dynamics, kinetic and structure-activity relationship studies.. Sci Rep..

[32] Khedkar SA, Malde AK, Coutinho EC, Srivastava S (2007). Pharmacophore modeling in drug discovery and development: an overview.. Med Chem..

[33] Mafethe O, Ntseane T, Dongola TH, Shonhai A, Gumede NJ, Mokoena F (2023). Pharmacophore Model-Based Virtual Screening Workflow for Discovery of Inhibitors Targeting Plasmodium falciparum Hsp90.. ACS Omega..

[34] Virtual Screening Workflow 2018-2 (2018). Glide version 6.6, LigPrep version 3.3,QikProp version 4.3..

[35] Shirley M (2018). Dacomitinib: First Global Approval.. Drugs..

[36] Kazandjian D, Blumenthal GM, Chen HY, He K, Patel M, Justice R (2014). FDA approval summary: crizotinib for the treatment of metastatic non-small cell lung cancer with anaplastic lymphoma kinase rearrangements.. Oncologist..

[37] Bolanowski M, Kaluzny M, Witek P, Jawiarczyk-Przybylowska A (2022). Pasireotide-a novel somatostatin receptor ligand after 20 years of use.. Rev Endocr Metab Disord..

[38] Yee D, Chavez J, Ruan W, Kleinberg D (2000). Inhibition of normal mammary gland development and breast cancer growth by IGFBP1. Proc 36th Annual Meeting of the American Society of Clinical Oncology..

[39] Smith J, Axelrod D, Singh B, Kleinberg D (2011). Prevention of breast cancer: the case for studying inhibition of IGF-1 actions.. Ann Oncol..

[40] Kuran D, Pogorzelska A, Wiktorska K (2020). Breast Cancer Prevention-Is there a Future for Sulforaphane and Its Analogs?. Nutrients..

[41] Ayoub NM, Al-Shami KM, Alqudah MA, Mhaidat NM (2017). Crizotinib, a MET inhibitor, inhibits growth, migration, and invasion of breast cancer cells in vitro and synergizes with chemotherapeutic agents.. Onco Targets Ther..

[42] Zhang J, Wang Y, Liu Z, Wang L, Yao Y, Liu Y (2021). Efficacy of dacomitinib in patients with EGFR-mutated NSCLC and brain metastases.. Thorac Cancer..

[43] Kalous O, Conklin D, Desai AJ, O'Brien NA, Ginther C, Anderson L (2012). Dacomitinib (PF-00299804), an irreversible Pan-HER inhibitor, inhibits proliferation of HER2-amplified breast cancer cell lines resistant to trastuzumab and lapatinib.. Mol Cancer Ther..

[44] Varga N, Smieško M, Jiang X, Jakob RP, Wagner B, Mühlethaler T (2024). Strengthening an Intramolecular Non‐Classical Hydrogen Bond to Get in Shape for Binding.. Angewandte Chemie..

[45] Niu M, Hu J, Wu S, Xiaoe Z, Xu H, Zhang Y (2014). Structural bioinformatics-based identification of EGFR inhibitor gefitinib as a putative lead compound for BACE.. Chem Biol Drug Des..

[46] Damghani T, Sedghamiz T, Sharifi S, Pirhadi S (2020). Critical c-Met-inhibitor interactions resolved from molecular dynamics simulations of different c-Met complexes.. J Molecular Structure..

[47] Timofeevski SL, McTigue MA, Ryan K, Cui J, Zou HY, Zhu JX (2009). Enzymatic characterization of c-Met receptor tyrosine kinase oncogenic mutants and kinetic studies with aminopyridine and triazolopyrazine inhibitors.. Biochemistry..

[48] Zhao Z, Xie L, Bourne PE (2019). Structural Insights into Characterizing Binding Sites in Epidermal Growth Factor Receptor Kinase Mutants.. J Chem Inf Model..

[49] Balogun TA, Ipinloju N, Abdullateef OT, Moses SI, Omoboyowa DA, James AC (2021). Computational Evaluation of Bioactive Compounds from Colocasia affinis Schott as a Novel EGFR Inhibitor for Cancer Treatment.. Cancer Inform..

[50] Engelman JA, Zejnullahu K, Gale CM, Lifshits E, Gonzales AJ, Shimamura T (2007). PF00299804, an irreversible pan-ERBB inhibitor, is effective in lung cancer models with EGFR and ERBB2 mutations that are resistant to gefitinib.. Cancer Res..

[51] Tomer KB, Crow FW, Gross ML, Kopple KD (1984). Fast atom bombardment combined with tandem mass spectrometry for the determination of cyclic peptides.. Anal Chem..

[52] Jia C, Qi W, He Z, Qiao B (2006). Sequencing peptides by electrospray ion-trap mass spectrometry: A useful tool in synthesis of Axinastatin 3.. Open Chemistry..

[53] Kalhor R, Reza MAS, Aali R, Abolhasani H, Mokhtarian MH, Kalhor H (2025). Next-generation bioremediation: Molecular decoding of fungal laccases for efficient degradation of bisphenol a and its derivatives.. Int J Biol Macromol..

[54] Collie GW, Michaelides IN, Embrey K, Stubbs CJ, Borjesson U, Dale IL (2021). Structural Basis for Targeting the Folded P-Loop Conformation of c-MET.. ACS Med Chem Lett..

[55] Songtawee N, Gleeson MP, Choowongkomon K (2013). Computational study of EGFR inhibition: molecular dynamics studies on the active and inactive protein conformations.. J Mol Model..

[56] E J, Liu Y, Guan S, Luo Z, Han F, Han W (2020). How Different Substitution Positions of F, Cl Atoms in Benzene Ring of 5-Methylpyrimidine Pyridine Derivatives Affect the Inhibition Ability of EGFR(L858R/T790M/C797S) Inhibitors: A Molecular Dynamics Simulation Study.. Molecules..

[57] Sohn J, Liu S, Parinyanitikul N, Lee J, Hortobagyi GN, Mills GB (2014). cMET Activation and EGFR-Directed Therapy Resistance in Triple-Negative Breast Cancer.. J Cancer..

