# Supplementary material for: Dual Inhibitors of c-MET and EGFR in Triple Negative Breast Cancer: Pharmacophore Modeling and Molecular Dynamics Based in Silico Drug Repositioning
Source: Iran J Pharm Res. 2025 Oct 24;24(1):e164183. doi: 10.5812/ijpr-164183 (PMC12606863; doi:10.5812/ijpr-164183)
Supplement: ijpr-24-1-164183-s001.pdf [file ijpr-24-1-164183-s001.pdf]

Dual inhibitors of c-MET and EGFR in triple negative breast cancer: pharmacophore modeling and molecular dynamics based *in silico* drug repositioning

Shadi Abkhiz[1], Parastoo Tarighi[1]*, Homa Azizian[2]*

[1] Department of Medical Biotechnology, Faculty of Allied Medical Sciences, Iran University of Medical Sciences, Tehran, Iran

[2] Department of Medicinal Chemistry, School of Pharmacy-International Campus, Iran University of Medical Science, Tehran, Iran

Table S1. pharmacophore model hypothesis and validation of c-Met active site

No.	Hypothesis	Phase Hypo Score	EF1%	BEDROC160.9	ROC
**1**	ARR_4	0.15	44.6	0.73	0.9
**2**	ARR_3	0.15	21.7	0.35	0.87
**3**	ARR_1	0.16	2.41	0.03	0.84
**4**	ARR_6	0.13	1.81	0.03	0.83
**5**	ARR_5	0.13	3.01	0.04	0.81
**6**	ARR_2	0.16	7.84	0.16	0.81
**7**	AAAR_5	0.13	1.21	0.03	0.71
**8**	AAR_2	0.15	7.23	0.14	0.7
**9**	AAR_1	0.15	0.6	0.01	0.66
**10**	AAAR_2	0.14	5.42	0.1	0.64
**11**	AARR_2	0.15	4.82	0.06	0.63
**12**	AAR_3	0.13	0.6	0.02	0.59
**13**	AAAR_7	0.12	1.21	0.02	0.58
**14**	AAR_4	0.13	1.81	0.02	0.57
**15**	AAAR_1	0.14	3.01	0.05	0.56
**16**	AARR_1	0.15	1.81	0.05	0.54
**17**	AAAR_6	0.13	0	0	0.51
**18**	AAAR_8	0.12	2.41	0.03	0.36
**19**	AAAR_4	0.14	3.62	0.05	0.27
**20**	AAAR_3	0.14	0	0	0.24

- ❖ (A) acceptor, (R) aromatic Ring

Table S2 pharmacophore model validation of EGFR

No.	Hypothesis	Phase Hypo Score	EF1%	BEDROC160.9	ROC
**1**	ADHHRRR_1	0.36	27.09	0.5	0.81
**2**	ADHHRRR_2	0.36	26.54	0.49	0.81
**3**	ADHHRRR_4	0.36	21.01	0.41	0.8
**4**	ADHHRRR_3	0.36	20.64	0.41	0.8
**5**	DDHHRRR_2	0.36	24.7	0.46	0.78
**6**	DDHHRRR_1	0.36	23.78	0.45	0.78
**7**	AHHRRR_2	0.31	21.56	0.42	0.75
**8**	AHHRRR_1	0.31	21.38	0.43	0.75
**9**	DHHHRRR_1	0.36	19.72	0.39	0.74
**10**	HRR_2	0.21	20.46	0.38	0.71
**11**	HRR_1	0.21	20.46	0.38	0.71
**12**	DHHRRR_2	0.31	17.14	0.35	0.69
**13**	DHHRRR_1	0.31	17.32	0.35	0.69
**14**	ADHHRR_2	0.31	20.09	0.4	0.68
**15**	ADHHRR_1	0.31	20.27	0.41	0.68
**16**	DDHHHRR_3	0.36	14.01	0.29	0.66
**17**	DDHHRR_4	0.32	13.27	0.26	0.65
**18**	DDHHRR_3	0.32	12.9	0.26	0.65
**19**	DDHHHRR_1	0.36	15.67	0.31	0.65
**20**	DDHHHRR_2	0.36	19.72	0.39	0.64
**21**	DDHHRR_2	0.32	16.96	0.34	0.6
**22**	DDHHRR_1	0.32	15.85	0.33	0.6
**23**	DHR_2	0.21	15.67	0.31	0.54
**24**	DHR_1	0.21	15.48	0.31	0.54
**25**	DHRR_2	0.24	16.59	0.34	0.54
**26**	DHRR_1	0.24	15.48	0.34	0.54
**27**	AHHRR_4	0.28	10.69	0.21	0.47
**28**	AHHRR_3	0.28	10.51	0.22	0.47
**29**	AHHRR_2	0.28	9.22	0.19	0.47
**30**	AHHRR_1	0.28	9.03	0.19	0.47
**31**	AHR_2	0.22	7.93	0.16	0.46
**32**	AHR_1	0.22	7.74	0.16	0.46
**33**	DHHRR_4	0.28	7.74	0.18	0.43
**34**	DHHRR_3	0.28	7.93	0.18	0.43
**35**	DHHRR_2	0.28	8.48	0.19	0.37
**36**	DHHRR_1	0.28	8.66	0.19	0.37
**37**	AHR_4	0.22	4.61	0.11	0.37
**38**	AHR_3	0.22	4.24	0.1	0.37
**39**	DDHHR_2	0.27	7.56	0.16	0.34
**40**	DDHHR_1	0.27	7.37	0.16	0.34

**41**	AHH_2	0.21	3.5	0.09	0.17
**42**	AHH_1	0.21	3.5	0.08	0.17
**43**	DHHR_4	0.24	5.34	0.12	0.14
**44**	DHHR_3	0.24	5.34	0.12	0.14
**45**	HHRR_2	0.24	7.56	0.16	0.13
**46**	HHRR_1	0.24	7.93	0.16	0.13
**47**	AHHR_2	0.24	5.53	0.11	0.08
**48**	AHHR_1	0.24	5.53	0.11	0.08
**49**	DHHR_2	0.24	3.69	0.08	0.06
**50**	DHHR_1	0.24	3.69	0.08	0.06

❖ (A) acceptor, (D) donor, (H) hydrophobic, (R) aromatic Ring

Table 3 c-MET pharmacophore-base virtual screening result

Number	Name	MMGBSA dG Bind (Kcal/mol)	Docking score	Glide energy
**1**	HEXACHLOROPHENE	-113.148	-10.1075	-46.1203
**2**	TEPOTINIB	-101.176	-11.7964	-69.1832
**3**	CRIZOTINIB	-99.4024	-12.6876	-47.2851
**4**	M TRICLABENDAZOLE	-94.9676	-9.85421	-51.5517
**5**	PASIREOTIDE	-93.793	-11.1342	-73.6724
**6**	CAPMATINIB	-88.8805	-11.6765	-56.9207
**7**	ENTRECTINIB	-88.5362	-9.65654	-56.936
**8**	TIRBANIBULIN	-87.2709	-8.83672	-51.4543
**9**	DACOMITINIB	-86.7629	-8.88709	-50.3116
**10**	VALRUBICIN	-81.411	-9.15353	-54.1269
**11**	M ENTRECTINIB	-78.1147	-11.4692	-49.9739
**12**	CARVEDILOL	-76.906	-9.4777	-52.6916
**13**	ONDANSETRON	-76.6241	-8.895	-45.0792
**14**	RIBOFLAVIN	-75.4588	-8.89034	-50.062
**15**	METHOTREXATE	-71.7786	-9.73011	-57.3134
**16**	PROPAFENONE	-71.5292	-10.0798	-48.2777
**17**	PRAZEPAM	-71.478	-9.3127	-29.9864
**18**	CHLORTHALIDONE	-69.1597	-11.5382	-39.6589
**19**	ASENAPINE	-69.0769	-8.90922	-29.0476
**20**	ZAFIRLUKAST	-67.1374	-9.14132	-62.3164
**21**	RALOXIFENE	-67.0197	-9.36132	-49.7611
**22**	DULOXETINE	-66.8535	-9.41139	-38.5964
**23**	VERICIGUAT	-66.7119	-9.59905	-50.6856
**24**	BEROTRALSTAT	-66.2648	-8.94213	-57.3349
**25**	PHENPROCOUMON	-66.1197	-10.6175	-37.175

**26**	MEBENDAZOLE	-65.4817	-10.5771	-40.4654
**27**	DICUMAROL	-64.5484	-8.83195	-28.309
**28**	M DOXEPIN	-64.2645	-8.92276	-27.9244
**29**	M FLURAZEPAM	-64.1209	-9.025	-33.533
**30**	CARBENICILLIN INDANYL	-63.6009	-9.93823	-45.5001
**31**	TOLCAPONE	-63.5186	-9.76391	-37.1588
**32**	HALAZEPAM	-63.4648	-9.18727	-31.5926
**33**	TEMAZEPAM	-62.7762	-9.20468	-42.078
**34**	ESLICARBAZEPINE ACETATE	-62.4287	-8.90296	-42.1344
**35**	METYRAPONE	-60.786	-9.25183	-35.6945
**36**	SAFINAMIDE	-59.9711	-9.66733	-43.0401
**37**	RIBOFLAVIN PHOSPHATE	-58.964	-8.94926	-32.7545
**38**	VEMURAFENIB	-57.7897	-9.39765	-42.2414
**39**	AZELASTINE	-57.0146	-9.48456	-42.1496
**40**	CLONAZEPAM	-56.4232	-8.83197	-35.4621
**41**	IMIQUIMOD	-56.4223	-9.23784	-32.6754
**42**	PEMIROLAST	-55.5068	-8.94433	-37.869
**43**	DOLUTEGRAVIR	-54.255	-9.0325	-36.7375
**44**	PHENYL AMINOSALICYLATE	-51.9899	-9.00876	-36.3829
**45**	SULFOXONE	-46.8798	-9.53568	-53.0929
**46**	FLUOXETINE	-45.337	-9.34561	-30.6715
**47**	DIPHENIDOL	-45.0924	-9.00331	-32.0155
**48**	M PICOSULFATE	-43.765	-10.634	-26.3706
**49**	IDELALISIB	-19.6456	-10.033	-33.4141

Table 4 EGFR pharmacophore-base virtual screening result

Number	Name	MMGBSA dG Bind (Kcal/mol)	Docking score	Glide energy
**1**	COBICISTAT	-111.8847622	-7.976882967	-65.24533701
**2**	BREMELANOTIDE	-109.0832434	-8.83930296	-82.35648537
**3**	LANREOTIDE	-108.9720011	-8.696933872	-82.68727303
**4**	SARALASIN	-106.0756078	-10.32767813	-84.88412094
**5**	PASIREOTIDE	-103.933776	-8.461799626	-87.40173244
**6**	ERLOTINIB	-102.8207575	-10.49881075	-58.32833481
**7**	AMPHOTERICIN B	-102.0083692	-9.227351036	-70.12491226
**8**	RITONAVIR	-100.432119	-8.063881863	-75.13867855
**9**	ANGIOTENSIN II	-97.08430091	-11.36072455	-90.97228622
**10**	OXYTOCIN	-95.27331343	-8.072898001	-75.14519882
**11**	DACOMITINIB	-94.05902222	-9.228156293	-53.19803667
**12**	SELPERCATINIB	-92.94007877	-7.773381954	-57.50364208

**13**	DESLANOSIDE	-91.72318869	-7.732376427	-60.40616035
**14**	AFATINIB	-90.75074083	-9.82760251	-57.31923389
**15**	ICOMTINIB	-90.60937593	-9.117567796	-46.54690838
**16**	SINCALIDE	-89.61580444	-8.475270446	-88.41709709
**17**	VALRUBICIN	-88.46690838	-8.106946764	-59.26667786
**18**	CANAGLIFLOZIN	-87.47979514	-7.969282841	-49.71697807
**19**	OSIMERTINIB	-87.31188282	-8.182380066	-53.84221077
**20**	SETMELANOTIDE	-86.54171478	-8.561830042	-76.67557907
**21**	TRAVOPROST	-86.46834304	-7.794164957	-50.48788548
**22**	SERTACONAZOLE	-86.26365295	-8.154896618	-42.26012397
**23**	GEFITINIB	-85.11110502	-8.294864547	-46.11164284
**24**	M FIDAXOMICIN	-84.91824415	-9.243739396	-61.97245979
**25**	OCTREOTIDE	-82.22650987	-9.081564314	-84.35085297
**26**	DESMOPRESSIN	-82.14994151	-7.627600218	-78.46639633
**27**	OLODATEROL	-81.44960446	-7.758166271	-48.76274395
**28**	IXAZOMIB	-80.30047283	-8.431593893	-50.24266434
**29**	ECONAZOLE	-79.97031509	-7.634420222	-44.12808692
**30**	EMPAGLIFLOZIN	-79.91250682	-8.978106311	-50.35771179
**31**	ERTUGLIFLOZIN	-79.46949357	-8.033418649	-50.24038696
**32**	LAROTRECTINIB	-79.47491552	-8.220617	-50.16515875
**33**	SELUMETINIB	-77.86457063	-9.280087318	-51.11791086
**34**	PEXIDARTINIB	-77.16376258	-8.358510518	-45.75952339
**35**	PITAVASTATIN	-77.11764822	-8.632883297	-47.93113804
**36**	LAPATINIB	-77.04849233	-8.046506427	-56.61333919
**37**	HYDROXYCHLOROQUINE	-76.37939638	-8.019644708	-37.39989996
**38**	M SELUMETINIB	-75.91977467	-8.549359981	-48.99003696
**39**	PLAZOMICIN	-75.47260511	-8.363491922	-57.04883385
**40**	DAPAGLIFLOZIN	-74.49088974	-8.075306783	-50.39474678
**41**	FENOLDOPAM	-72.83264489	-8.516723004	-34.35267067
**42**	BORTEZOMIB	-72.6139127	-8.136782142	-53.10796928
**43**	BINIMETINIB	-72.40349303	-8.787672571	-50.09866142
**44**	DOCETAXEL	-71.92674407	-7.867561698	-58.42463207
**45**	IDARUBICIN	-70.74819383	-7.963606869	-53.76989174
**46**	EZETIMIBE	-70.61299726	-7.810521292	-43.17826748
**47**	ESTETROL	-68.3399576	-7.961487054	-38.75888538
**48**	SACUBITRIL	-68.29263184	-8.269772287	-47.37074089
**49**	CRIZOTINIB	-67.49959519	-10.78915313	-45.30870056
**50**	ROSUVASTATIN	-66.7910999	-8.209282505	-47.39077997
**51**	OMADACYCLINE	-66.2954344	-7.602046971	-38.16872978
**52**	WARFARIN	-66.00394913	-7.656521728	-41.11967039
**53**	CEFOPERAZONE	-65.71877405	-8.325124185	-69.53261042

**54**	ARBUTAMINE	-65.47037292	-8.079256015	-43.62244034
**55**	ATORVASTATIN	-64.64547348	-7.611603783	-51.82890272
**56**	PRALATREXATE	-64.59931054	-7.568376666	-56.067626
**57**	REGADENOSON	-64.26434974	-7.934380892	-54.4534111
**58**	CHLOROQUINE	-64.19082407	-8.00233595	-34.40816665
**59**	PHENPROCOUMON	-64.02186948	-7.607243222	-33.30106926
**60**	EPIRUBICIN	-64.00040011	-7.928747887	-57.68253136
**61**	APOMORPHINE	-63.81873803	-7.898668281	-32.39686489
**62**	M AMODIAQUINE	-63.3886118	-8.8407572	-42.32088518
**63**	CANGRELOR	-62.20120826	-8.408537228	-63.51173592
**64**	MEBENDAZOLE	-61.95913863	-7.581162226	-36.42149496
**65**	RIBOFLAVIN	-61.53852146	-9.768021113	-51.4049263
**66**	TADALAFIL	-61.49903723	-7.940735817	-31.92233968
**67**	METHACYCLINE	-61.11560515	-7.792014848	-46.65385818
**68**	TOLCAPONE	-60.95863285	-7.896705338	-36.77293456
**69**	ERAVACYCLINE	-59.36395404	-8.117702737	-53.65415382
**70**	DABIGATRAN	-58.07413961	-7.755062958	-53.71243477
**71**	CERIVASTATIN	-57.92761423	-7.879352339	-46.05922318
**72**	OXYTETRACYCLINE	-57.71911568	-7.969929483	-48.08411407
**73**	M REMDESIVIR	-57.63974881	-8.817434312	-36.11153984
**74**	MASOPROCOL	-57.52373866	-9.310321122	-35.24548817
**75**	VALGANCICLOVIR	-56.85275931	-8.020737212	-48.0977807
**76**	RIBOFLAVIN PHOSPHATE	-55.80884545	-8.404988443	-49.53312206
**77**	CRISABOROLE	-53.5106133	-8.143863446	-32.8743372
**78**	CLADRIBINE	-52.59113705	-8.392390387	-34.20452642
**79**	TRIAMTERENE	-52.38548341	-8.886456723	-32.49963605
**80**	SULFAPHENAZOLE	-52.02587905	-8.016733319	-37.24487448
**81**	CLOFARABINE	-51.94497088	-7.685688932	-33.64342761
**82**	SULFADOXINE	-48.54466733	-7.821031995	-38.59629107
**83**	ADENOSINE	-47.59422827	-9.583722275	-36.02366257
**84**	VIDARABINE	-46.34919229	-9.005628955	-41.20659637
**85**	LEVOMEFOLATE	-44.063244	-8.040880749	-43.04594088
**86**	FLUDARABINE	-40.61962167	-7.716735678	-38.65628529
**87**	M PENCICLOVIR	-37.95043983	-7.817235006	-49.87418365

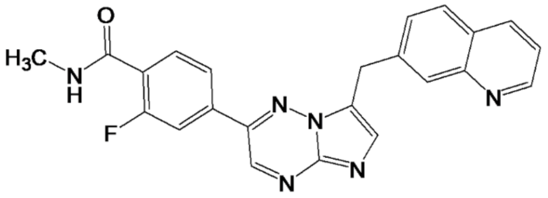

Cabmatinib

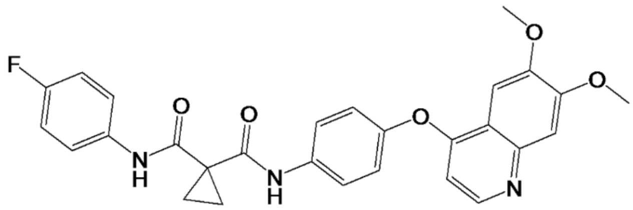

Cabozatinib

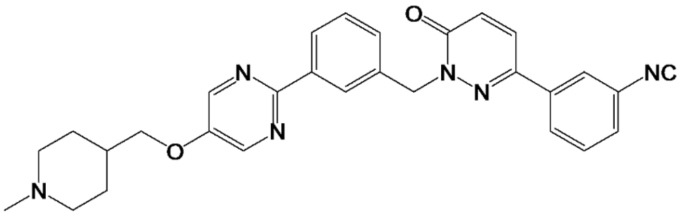

Tepotinib

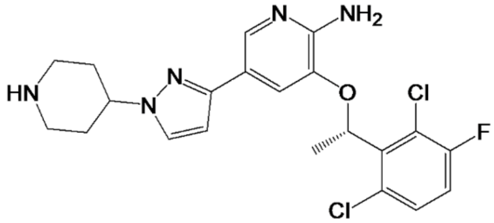

Crizotinib

Figure 1. 2D representation of c-MET FDA-approved inhibitors

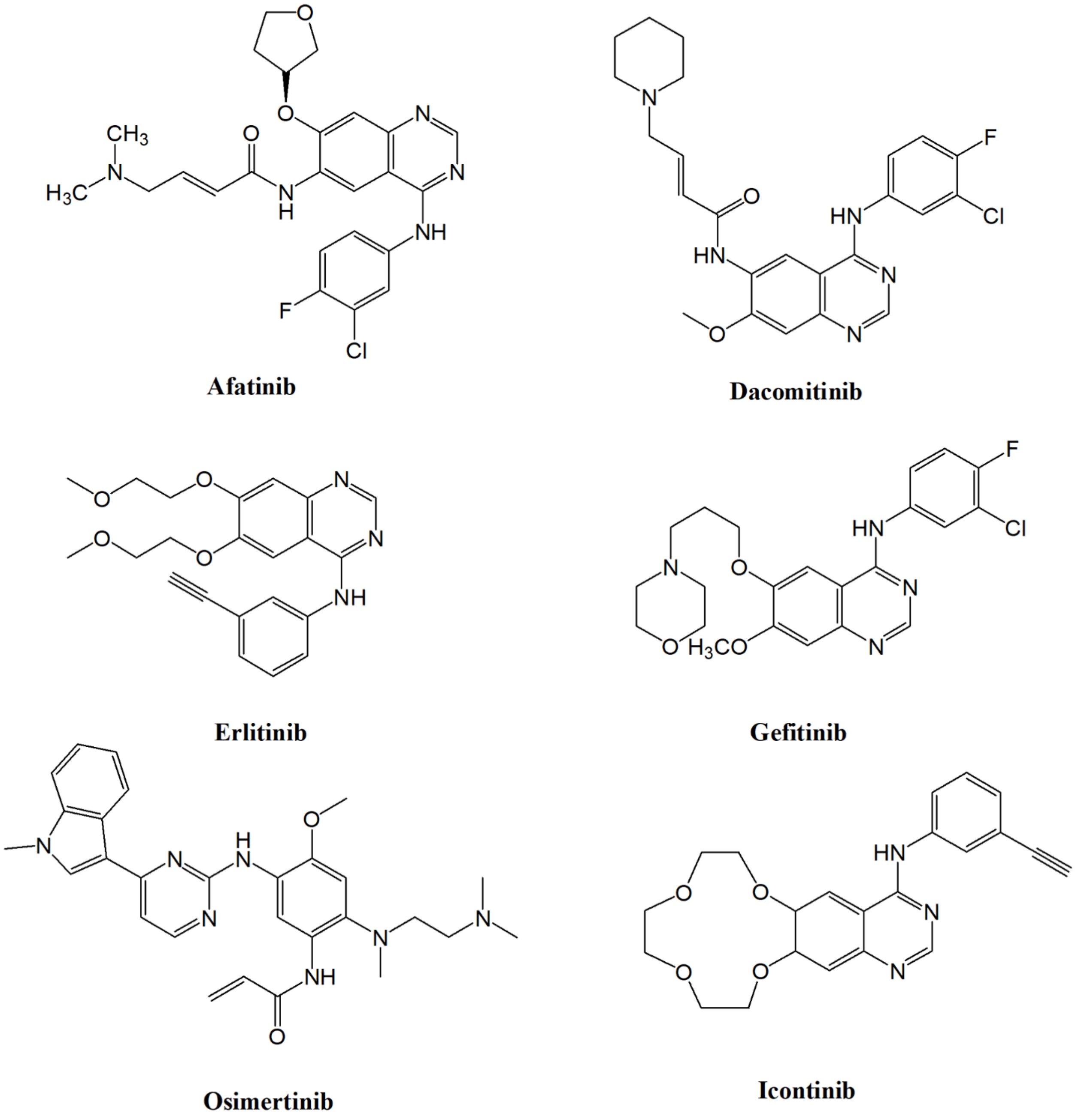

Figure 2. 2D representation of EGFR FDA-approved inhibitors

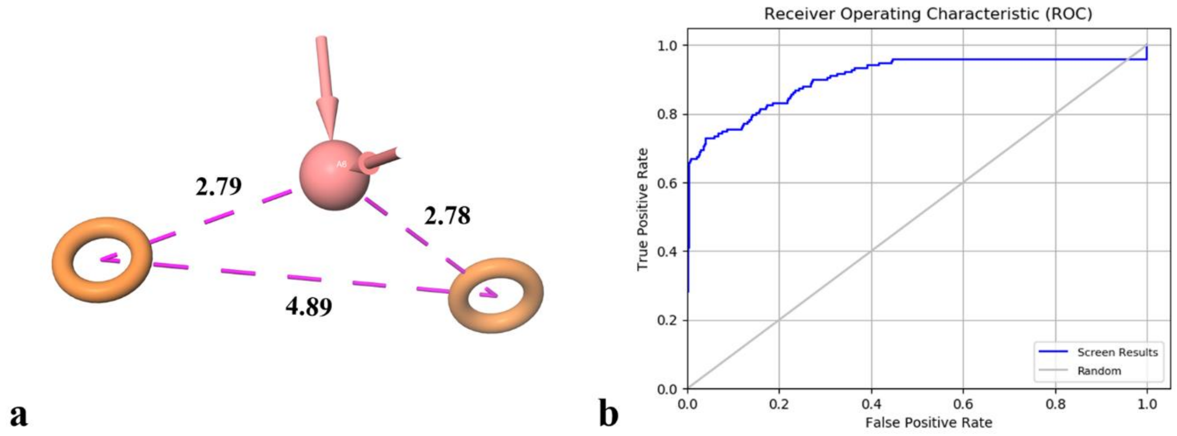

Figure 3. 3D representation of the highest ranked pharmacophore hypothesis of c-MET, ARR_4. This figure shows the ARR_4 inter-site positions (acceptor pink, aromatic ring orange) and distances in angstroms (a), along with the relative ROC (b)

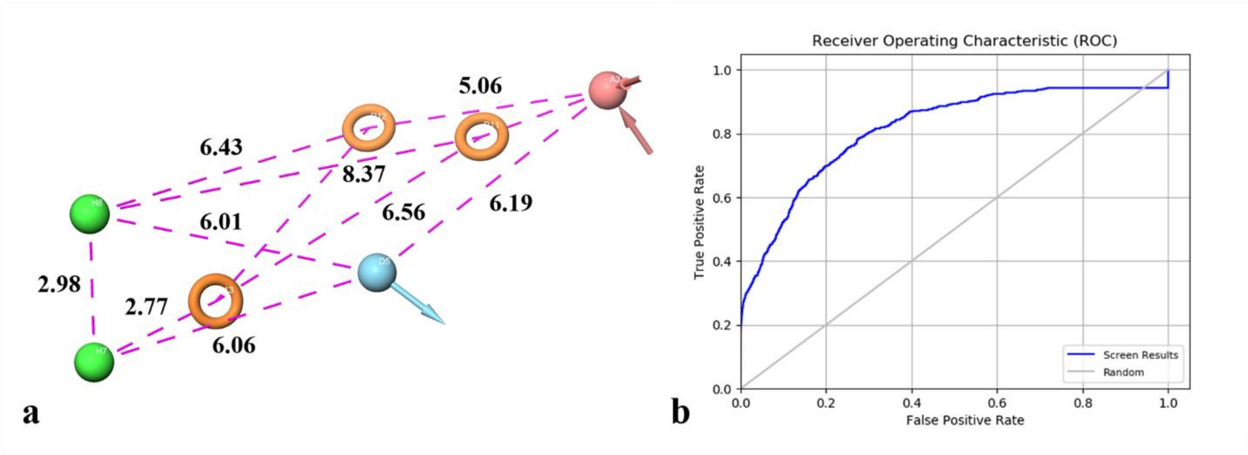

Figure 4. ADHHRRR_1 is the highest ranked pharmacophore hypothesis for selected EGFR. This figure presents the ADHHRRR_1 inter-site position (acceptor pink, aromatic ring orange, donor blue, and hydrophobic green) and distances in angstroms (a). Furthermore, (b) exhibits the ROC curve for this pharmacophore hypothesis.

Table 5. The binding energy calculation, residues and type of interactions over the c-MET active site through induced fit docking procedure

No.	Name	MM-GBSA ΔG_{Bind} (Kcal/mol)	Interaction over c-MET active site		
			H-Bond	Hydrophobic	Electrostatic

1	Crizotinib	-99.4024	Met1160, Asp1222, Tyr1159, Pro1158, Lys1161	Tyr1230, Ala1221, Ala1226, Val1092, Lys1110, Leu1157, Leu1140, Met1211, Ile1084, Ala1108	-
2	Pasireotide	-93.793	Arg1086, Met1160, His1162, Asp1164, Glu1233, Ile1084, Gly1163, Pro1158	Ile1084, Tyr1159, Tyr1230, Ala1108 Val1092	-
3	Dacomitinib	-86.7629	Met1160, Tyr1159, Arg1208	Met1211, Tyr1230, Arg1086, Ala1108, Ile1084, Val1092, Tyr1159, Ala1221, Leu1157, Met1160	-
4	Valrubicin	-81.411	Val1083	Ala1221, Met1211, Tyr1230	-
5	Riboflavin	-75.4588	Met1160, Ile1084, Arg1208, Asp1164	Leu1140, Leu1157, Met1211, Tyr1230, Ile1084, Val1092, Ala1108	-
6	Phenprocoumon	-66.1197	Met1160, Pro1158	Tyr1230, Tyr1159, Ala1226, Val1092, Lys1110, Leu1157, Ala1108, Met1160, Met1211, Ala1221, Ile1084	-
7	Mebendazole	-65.4817	Met1160, Ile1084, Lys1161	Tyr1230, Tyr1159, Ile1084, Ala1108, Val1092, Ala1221, Ala1226	-
8	Tolcapone	-63.5186	Met1160, Pro1158, Gly1085, Tyr1159	Met1211, Tyr1230, Ala1221, Ala1226, Leu1140, Ile1084, Val1092, Ala1108	Tyr1159

Table 6. The binding energy calculation, residues and type of interactions over the EGFR active site through induced fit docking procedure

No.	Name	MM-GBSA ΔG_{Bind} (Kcal/mol)	Interaction over EGFR active site		
			H-Bond	Hydrophobic	Electrostatic
1	Pasireotide	-103.9337	Cys797, Asp855, Asn842, Asp800 Val717- Glu804, Phe795, Leu718, Gly796	Leu718, Gly719, Arg841, Val717- Val726- Ala743, Leu844, Lys745	
2	Dacomitinib	-94.0590	Met793, Gly719-Gln791	Leu718, Lys745, Leu788, Ala743, Met793, Leu844,	-
3	Valrubicin	-88.4669	Lys745, Cys797, Asp800, Gly719, Met793	Lys745, Val726, Leu844, Leu747	Asp855
4	Crizotinib	-67.49959	Met793, Asp800, Gln791, Leu718	Ala743, Val726, Lys745, Leu718, Leu844	-
5	Phenprocoumon	-64.0219	-	Lys745, Val726, Ala743, Leu844, Leu718,	-
6	Mebendazole	-61.9591	Met793, Gly796, Pro794	Leu718, Ala743, Leu844, Val726	-
7	Riboflavin	-61.5385	Met793, Cys797, Leu718- Leu792	Leu718, Leu747, Lys745, Val726, Ala743, Leu844,	-

8	Tolcapone	-60.9586	Met793, Gln791	Lys745, Met766, Leu788, Leu718, Ala743, Leu844	-

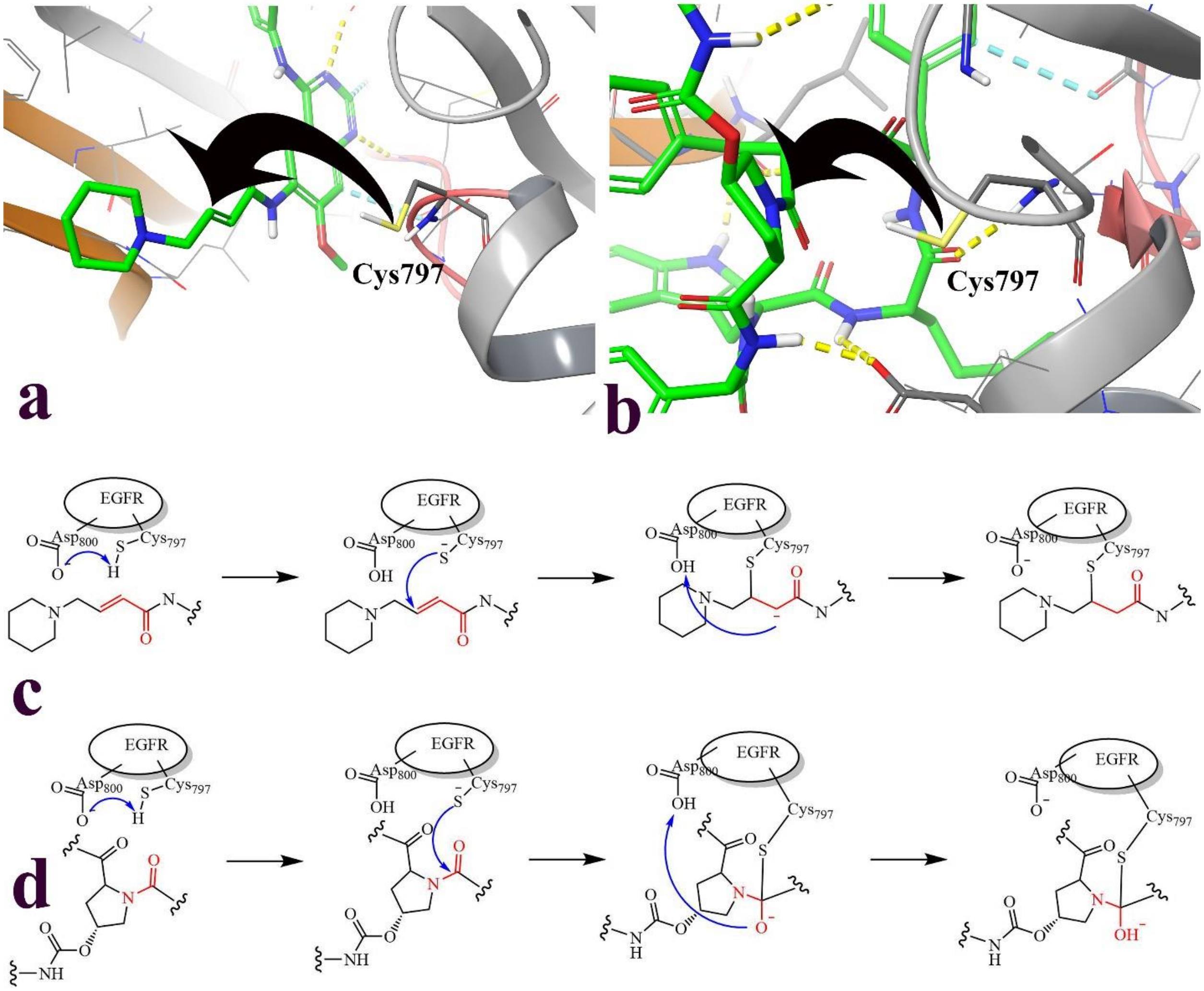

Figure 5. 3D representation of spatial arrangement of the (piperidin-1-yl) but-2-enoyl tail of dacomitinib (a) and the modified L-hydroxy proline moiety of pasireotide (b) around the Cys797 of the kinase hinge region (the nucleophile and the probable electrophilic center defined by the head and tail of the black arrows). The detailed mechanism of the proposed nucleophilic-electrophilic reaction possibility by dacomitinib and pasireotide represented in part (c) and (d), respectively.

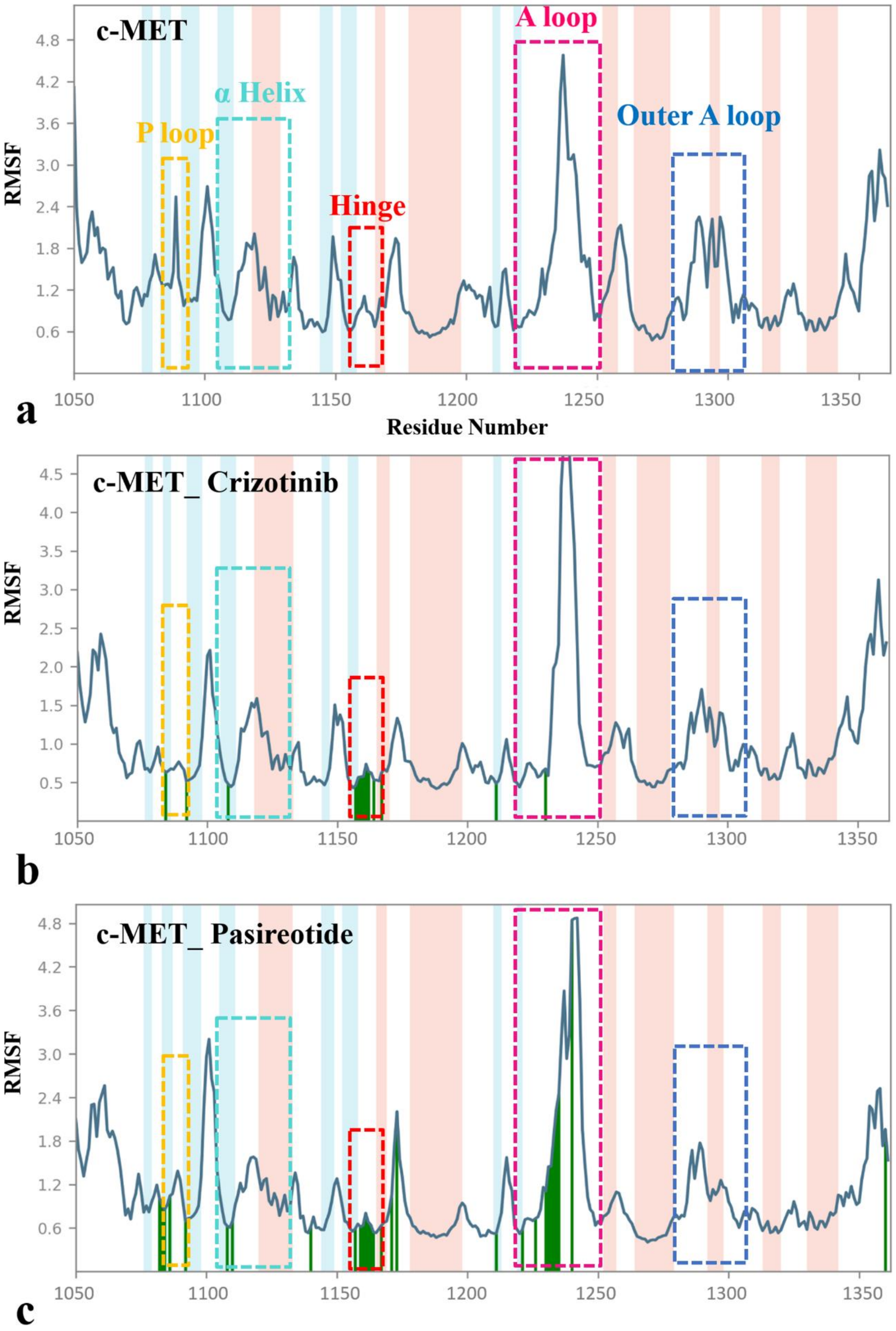

c-MET
c-MET_ Crizotinib
c-MET_ Pasireotide
P loop
α Helix
Hinge
A loop
Outer A loop
RMSF
Residue Number
a
b
c

Figure 6. RMSF plot of c-MET residue in non-bonded-state (a) in complexed with crizotinib (b) and in complex with pasireotide (c) over 80 ns MD simulation time. α-helical and ß-strand regions are highlighted by pink and blue backgrounds, respectively.

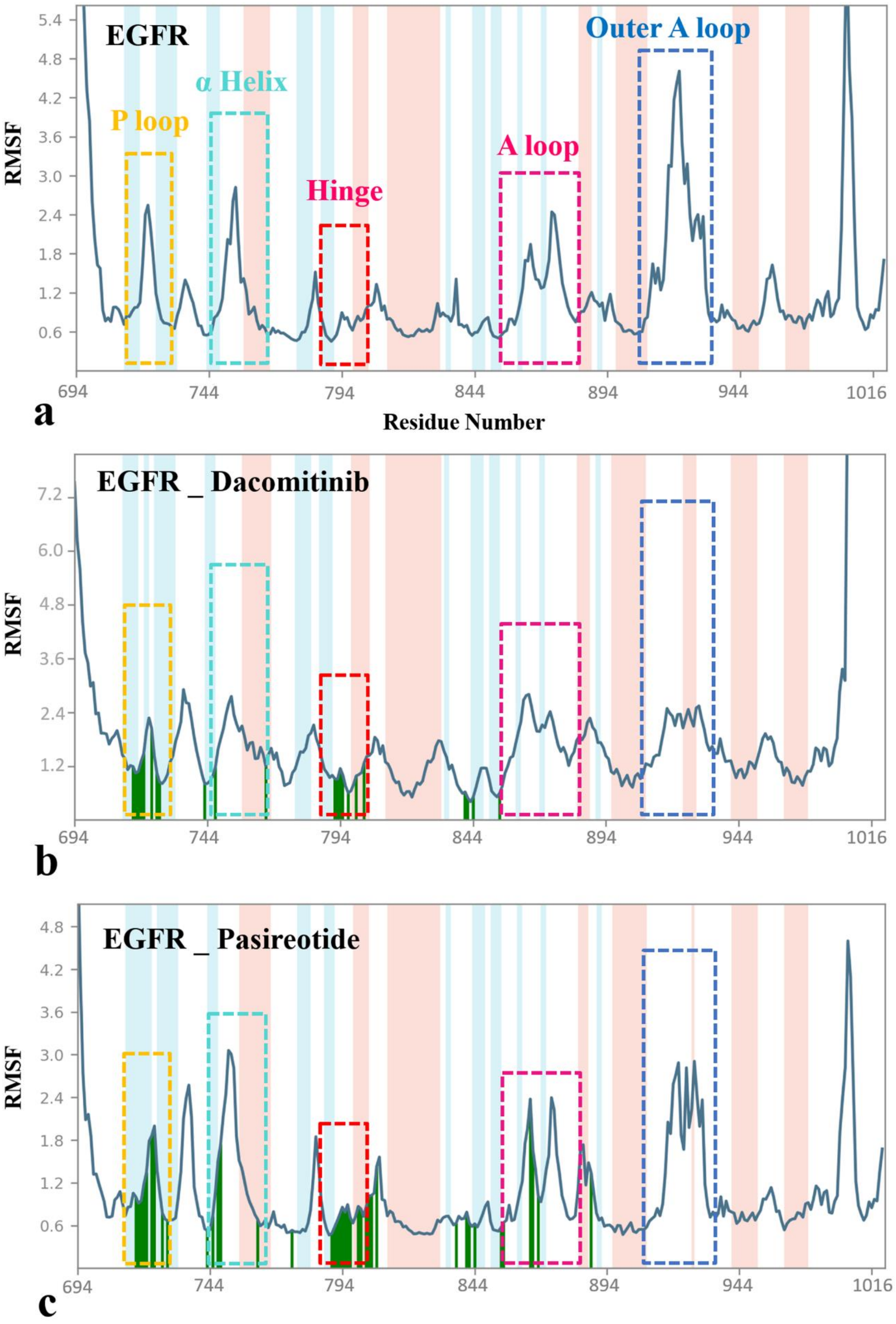
EGFR
Outer A loop
α Helix
P loop
A loop
Hinge
RMSF
Residue Number
a
EGFR _ Dacomitinib
b
EGFR _ Pasireotide
c
694
744
794
844
894
944
1016

Figure 7. RMSF plot of EGFR kinase domain residue in non-bonded-state (a) in complexed with dacomitinib (b) and in complex with pasireotide (c) over 80 ns MD simulation time. α-helical and ß-strand regions are highlighted by pink and blue backgrounds, respectively.

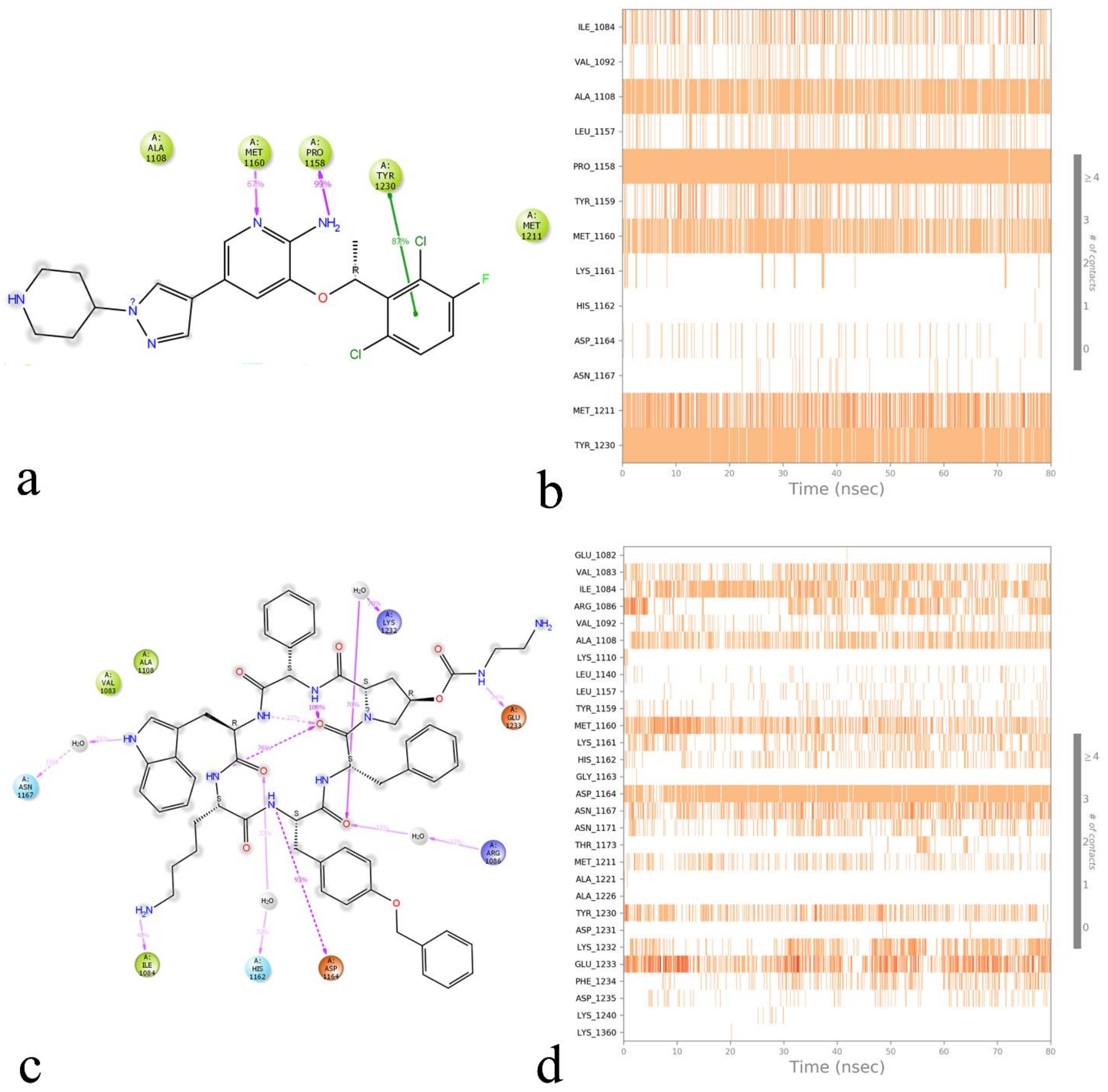

Figure 8. Detailed interactions between crizotinib (a) and pasireotide (c) over c-MET active site residues in the selected trajectory are shown in the schematic which is responsible for over 30% of MD simulation time. The timeline renderings of interacting residues over the whole simulation time of c-MET complexed with crizotinib (b) and pasireotide (d), respectively.

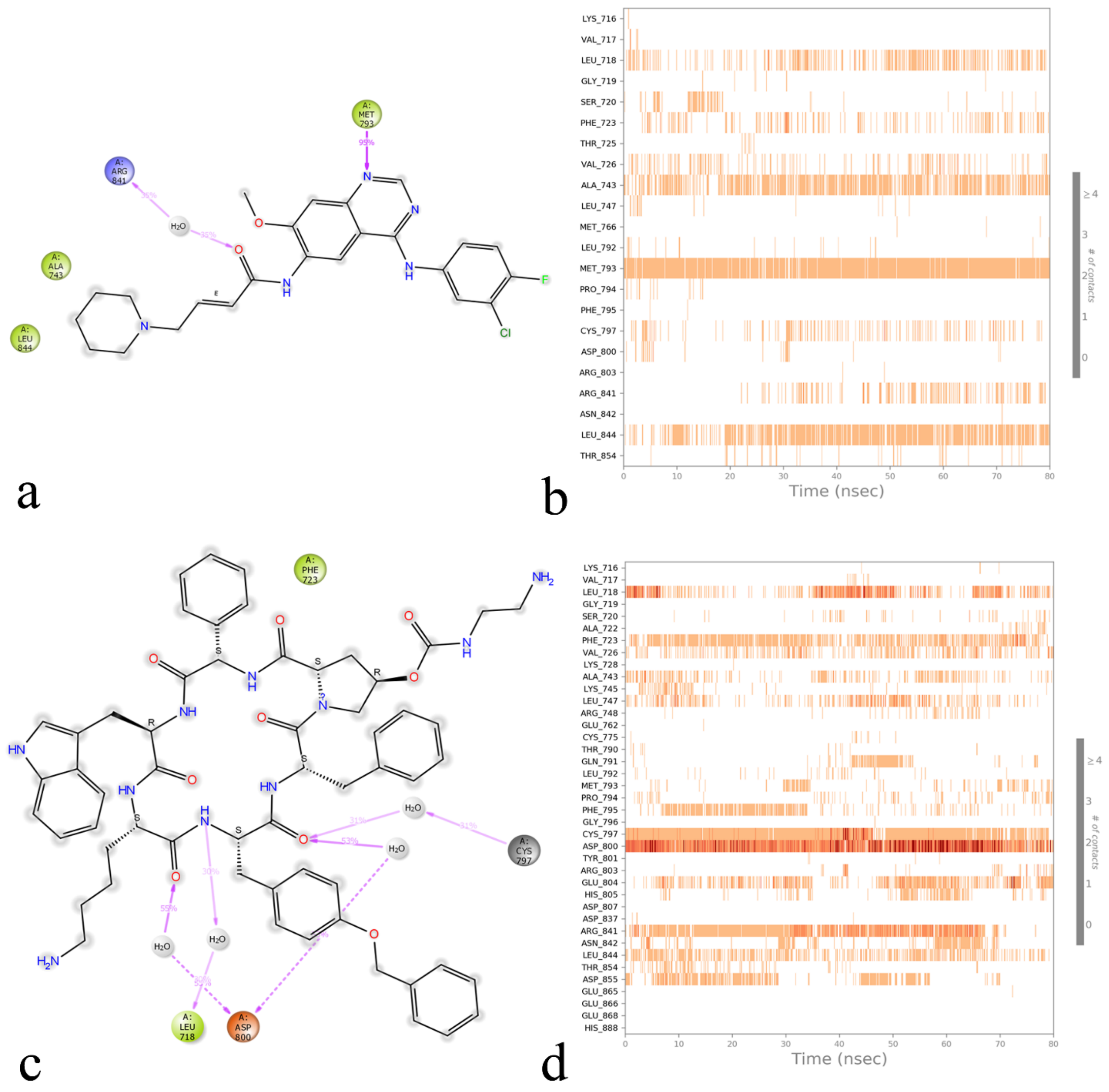

Figure 9. Detailed interactions between dacomitinib (a) and pasireotide (c) over EGFR active site residues in the selected trajectory are shown in the schematic which is responsible for over 30% of MD simulation time. The timeline renderings of interacting residues over the whole simulation time of EGFR complexed with dacomitinib (b) and pasireotide (d), respectively.

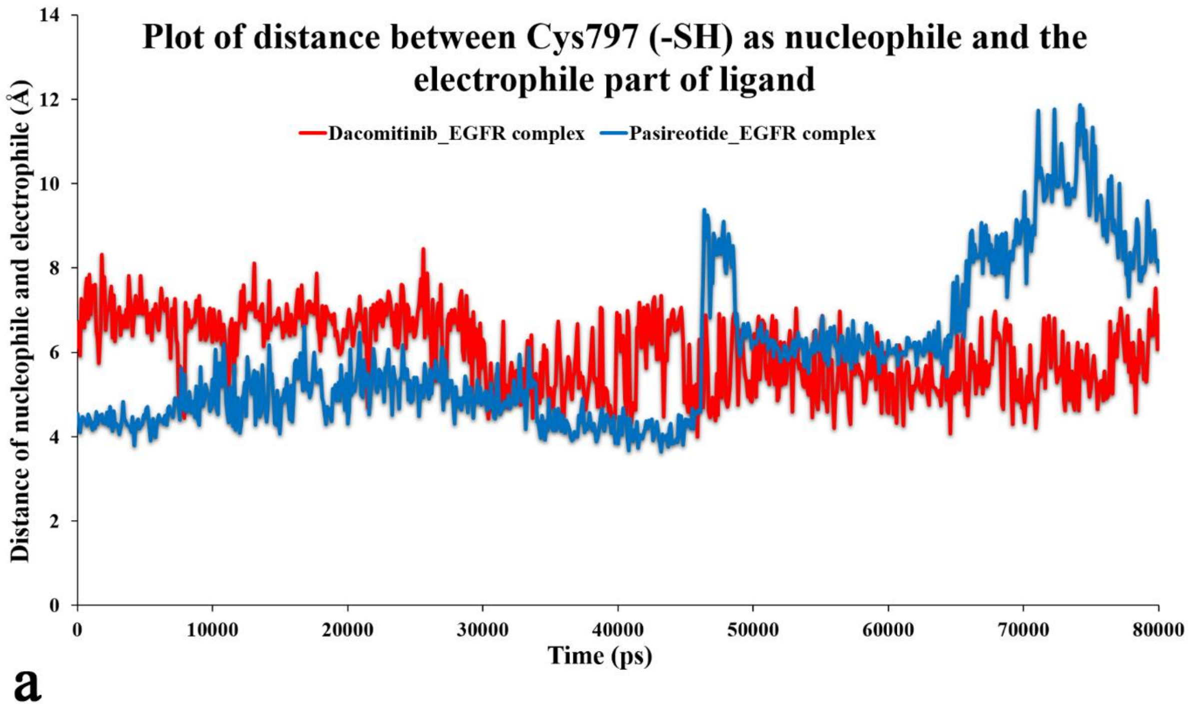

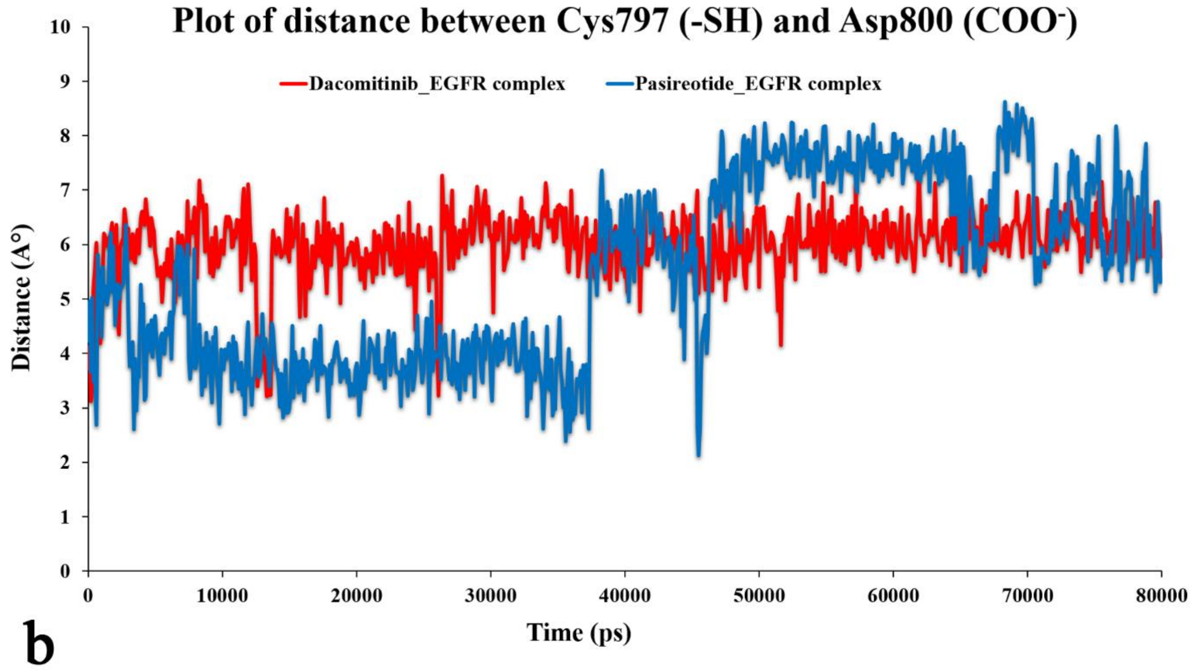

Figure 10. Plot of the distance between Cys797 (-SH) as nucleophile at the hinge region of EGFR kinase domain with the electrophile center of dacomitinib (in red color) and pasireotide (in blue color) (a), and the Plot of the distance between Cys797 (-SH) as nucleophile and Asp800 (COO^-) as base

Table 7 Binding free energy (ΔGbind) values

Complex name	ΔG_{bind} Kcal/mol	Solvation effects Kcal/mol	Van der waals energy Kcal/mol	Electrostatic energy Kcal/mol
c-MET_Pasireotide	-58.22	-1592.92	-2958.971	-1682.530
c-MET_Crizotinib	-77.56	-2136.35	-2958.971	-1682.530
EGFR_Pasireotide	-84.37	-1648.90	-2829.229	-1682.212
EGFR_Dacomitinib	-94.06	-1635.35	-2874.917	-1671.286

Table 8 Proposed potential c-MET and EGFR dual inhibitor, mechanism of action and therapeutic indication. The information is derived from drug bank (https://www.Drugbank.ca).

**No.**	**Name (DB No.)**	**Mechanism of Action**	**Indication**	**FDA-approved year**
1.	PASIREOTIDE (DB06663)	Activates somatostatin receptors	treatment of Cushing's disease	2012
2.	VALRUBICIN (DB00385)	it inhibits the incorporation of nucleosides into nucleic acids, causes extensive chromosomal damage, and arrests cell cycle in G2	treatment of BCG-resistant bladder carcinoma	1998
3.	DACOMITINIB (DB11963)	inhibitor of the human epidermal growth factor receptor (EGFR) family (EGFR/HER1, HER2, and HER4) tyrosine kinases.	Treatment of non-small cell lung cancer (NSCLC)	2018
	RIBOFLAVIN (DB00140)	Binds to riboflavin hydrogenase, riboflavin kinase, and riboflavin synthase	used to correct vitamin B2 deficiency	

	CRIZOTINIB (DB08865)	inhibitor of tyrosine kinase, anaplastic lymphoma kinase (ALK), hepatocyte growth factor receptor (HGFR, c-MET), ROS1 (c-ros), and Recepteur d'Origine Nantais (RON)	Treatment of metastatic non-small cell lung cancer (NSCLC) and treatment of systemic anaplastic large cell lymphoma (ALCL)	2011
	MEBENDAZOLE (DB00643)	binding to the colchicine-sensitive site of tubulin, thus inhibiting its polymerization or assembly into microtubules.	treat helminth infections	1974
	PHENPROCOUMON (DB00946)	anticoagulant drug, inhibits vitamin K reductase	prevention and treatment of thromboembolic disease	
	TOLCAPONE (DB00323)	inhibitor of catechol-O-methyltransferase (COMT)	treatment of Parkinson's Disease	1998

1DB no.: Drug Bank Accession Number
